# Therapeutic potential of ginseng and ginsenosides on muscle wasting disorders: Cachexia, sarcopenia, anorexia, and aging

**DOI:** 10.1016/j.jgr.2026.101021

**Published:** 2026-03-17

**Authors:** Chang-Hwan Bae, Subramanian Muthamil, Ji Hyo Lyu, Jong Min Oh, Ung Cheol Shin, Maic Audo Eybi Mayer Sihombing, Thi Quynh Dan Nguyen, Seon-Wook Kim, Jong-Hoon Kim, Md M.N. Azim, Jun Hong Park

**Affiliations:** a Herbal Medicine Resources Research Center, Korea Institute of Oriental Medicine, Naju, 58245, Republic of Korea; b University of Science & Technology (UST), KIOM campus, Korean Convergence Medicine Major, Daejeon, 34054, Republic of Korea; c Department of Anatomy, College of Medicine, Korea University, Seoul, 02841, Republic of Korea; d Department of Physiology, College of Veterinary Medicine, Jeonbuk National University, Iksan, 54596, Republic of Korea; e Biosafety Research Institute, College of Veterinary Medicine, Jeonbuk National University, Iksan 54596, Republic of Korea

**Keywords:** Muscle wasting, Cachexia, Sarcopenia, Ginseng, Ginsenosides, Anorexia, Aging, HIF-1

## Abstract

Muscle wasting disorders mainly arise from appetite loss, chronic diseases, prolonged immobilization, and aging. Chronic diseases such as cancer, stroke, neurological diseases, heart and kidney dysfunction are the primary reasons for muscle mass loss. Muscle atrophy caused by an imbalance between protein synthesis and degradation in myocytes and the severe muscle-wasting conditions include cachexia, sarcopenia, anorexia, and fatigue. Cachexia is characterized by loss of >5% of body weight or a body mass index (BMI) < 20 kg/m^2^. Sarcopenia is characterized by age-associated declines in muscle mass, strength, and gait speed, while anorexia refers to weight loss associated with dietary restriction. Recent evidence suggests that the HIF-1 pathway plays an important role in muscle wasting disorders. Given the significant health burden caused by muscle wasting, there is an urgent need to identify novel biomarkers for clinical diagnosis and to develop effective therapeutic strategies. In this context, plant-derived compounds, particularly ginseng and its bioactive constituent, ginsenosides, have recently been explored for their potential in mitigating muscle wasting conditions. This review provides an updated overview of current research on the therapeutic applications of ginseng and ginsenosides in mitigating muscle atrophy-related conditions, including cachexia, sarcopenia, fatigue, anorexia, aging, and obesity.

## Graphical abstract

**Figure:**
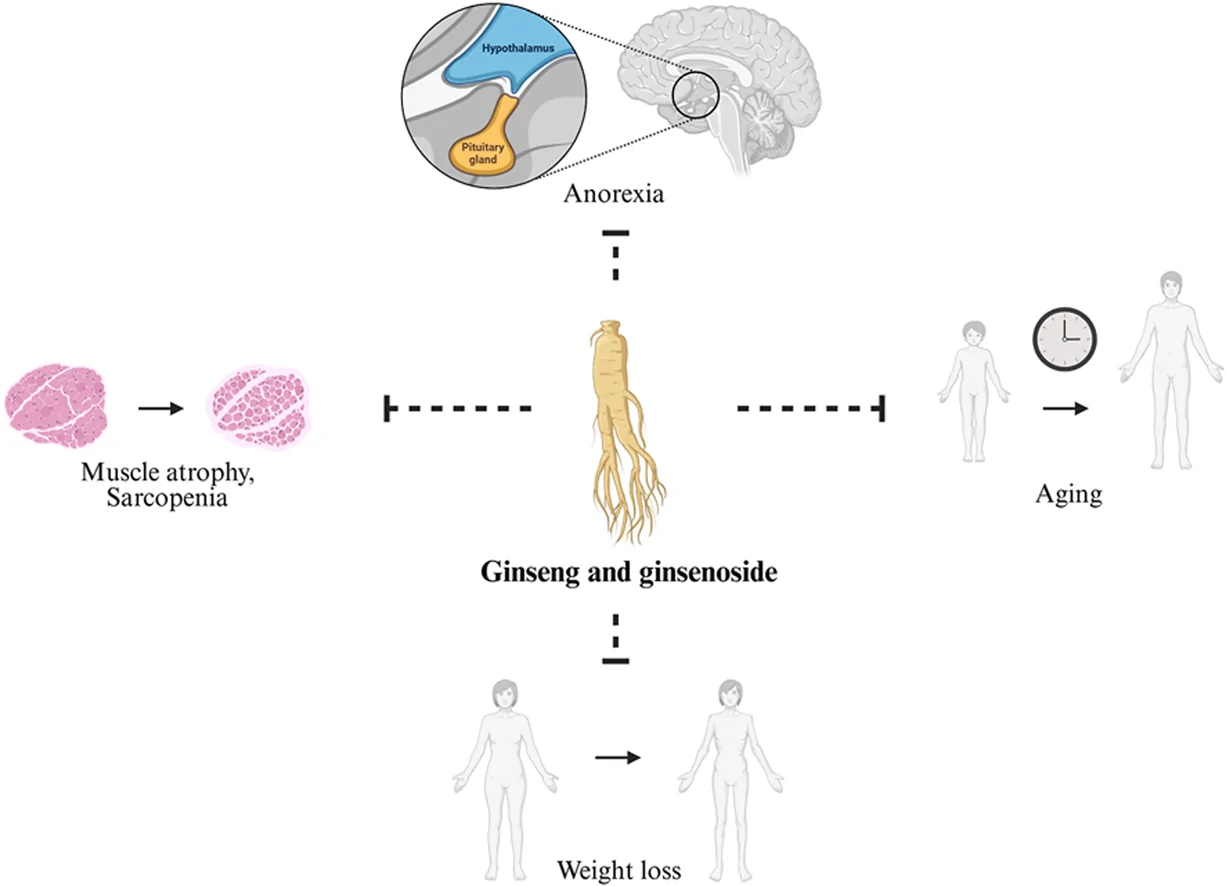

## 1 Introduction

Skeletal muscle is a highly adaptable and dynamic tissue, comprising approximately 30–40% of total body mass. It is essential for various physiological processes, including movement, respiration, and serves as a reservoir for biomolecules [1]. Muscle atrophy, defined as the loss or deterioration of muscle tissue, can arise from various causes including prolonged inactivity, neurogenic disorders, aging, malnutrition, muscle disuse, and diseases affecting the musculoskeletal and nervous systems. This condition is primarily driven by an imbalance between protein synthesis and breakdown [2]. Key pathways involved in muscle degradation include the ubiquitin–proteasome system (UPS), apoptosis, and autophagy [3]. Muscle atrophy also activates multiple signaling cascades, leading to transcriptomic and proteomic alterations, which affect both protein synthesis and degradation [4]. Major regulatory pathways of skeletal muscle mass include the insulin/IGF1-Akt-mTOR, TGF-β/myostatin/activin/BMP, and β-adrenergic signaling pathways [[1], [2], [3], [4]]. The complexity of these mechanisms presents challenges in developing effective treatments for muscle wasting.

Muscle atrophy is associated with several chronic conditions, including heart failure, cachexia, sarcopenia, anorexia, diabetes, injuries, malnutrition, obesity, aging, and extended periods of immobilization [3]. Cachexia, particularly cancer-associated cachexia (CAC), is characterized by a progressive loss of skeletal muscle that cannot be fully reversed through nutritional support. It affects over 70% of cancer patients and is also observed in chronic illnesses such as AIDS, kidney disease, heart failure, and chronic obstructive pulmonary disease [5].

Sarcopenia is defined as an age-related decline in skeletal muscle mass and strength. According to the Society of Sarcopenia, Cachexia, and Wasting Disorders, its diagnosis is based on reduced mobility, evaluated through walking speed and muscle mass assessments. Sarcopenia is a condition of significant reduction in the size and number of muscle fibers, particularly fast-twitch fibers, alongside increased infiltration of fibrotic and adipose tissue within muscles. While aging-related biological changes are the primary cause, factors such as obesity and excessive fat accumulation in skeletal muscle significantly accelerate its progression [6]. Additional contributors include inflammation, mitochondrial dysfunction, neuromuscular junction degradation, satellite cell numbers reduction, and hormonal imbalances. Molecular pathways implicated in sarcopenia include TGF-β signaling, apoptosis, and mitochondrial dysfunction [6].

Anorexia nervosa is a psychiatric eating disorder characterized by an extreme fear of gaining weight, distorted body image, and severe dietary restrictions. Previous studies indicate that anorexia induces structural and functional changes in skeletal muscle [7]. Although its exact causes remain unclear, genetic and hormonal influences, social factors, anxiety disorders, and early childhood eating habits play contributory roles. The prevalence of anorexia ranges from 0.3% to 4% among males and females, with onset typically occurring between the ages of 14 and 17. It carries the highest mortality rate among psychiatric disorders, with suicide being a leading cause of death [8].

Aging is another major factor contributing to skeletal muscle decline, leading to frailty, increased risk of chronic illnesses, obesity, and poor nutritional habits. In-depth research indicates that muscle mass decreases annually by approximately 0.64–0.98% in women and 0.8–0.98% in men, whereas muscle function declines more rapidly, by 2.5–3% per year in women and 3–4% per year in men [9].

Above all, obesity is a global public health concern affecting individuals across all age groups. It is closely linked to conditions such as cardiovascular disease, type 2 diabetes, coronary heart disease, cancer, stroke, hypertension, and other lifestyle factors [10]. While sarcopenia in older adults is well documented, obesity-related muscle loss remains less explored. Obesity-related muscle wasting is associated with poor psychological well-being, diminished quality of life, and comorbidities such as type 2 diabetes and hypertension [11]. Furthermore, obesity exacerbates muscle protein imbalance and anabolic resistance, leading to reduced physical activity, lower lean mass proportion, intracellular lipotoxicity, impaired muscle regeneration, chronic inflammation, insulin resistance, and endocrine dysfunction. Obesity also contributes to skeletal muscle remodeling and mitochondrial apoptosis. According to the World Health Organization, the global prevalence of obesity has risen sharply, contributing to a substantial increase in body weight-related health complications [12].

With this background, ginseng has been widely used in traditional herbal medicine for centuries, particularly in China, Korea, Japan, as well as in Western countries. Historical records indicate its therapeutic use for over 2000 years, with traditional oriental medicine describing its pharmacological benefits, including anti-fatigue, immune-boosting, and nourishing properties [13]. Ginseng is a slow-growing plant with fleshy roots, classified into American *Panax quinquefolius* and Asian *Panax ginseng* varieties based on their bioactive compounds. Research has demonstrated a wide range of pharmacological effects, including antioxidant, anti-inflammatory, cognitive-enhancing, and immune-modulating activities, and potential therapeutic applications in diabetes, erectile dysfunction, fatigue, and the mitigation of chemotherapy-induced side effects. The most common ginsenosides and their effect on cancer, muscle wasting, inflammation, apoptosis, and aging are listed in Table 1. Short-term supplementation with American ginseng helps to reduce exercise-induced muscle damage by lowering lipid peroxidation and inflammatory markers [14]. Similarly, *P*. *ginseng* root extract has been shown to mitigate lipopolysaccharide-induced inflammation, cytokine release, and oxidative stress, which may help prevent mitochondrial dysfunction in macrophages and adipocytes [15].

**Table 1:** Summary of the therapeutic effect of key ginseng components.

Therapeutical effect	Associated ginsenosides	Detail	References
1. Muscle protective effect	Rg1, Rb1, Rd, Rg3, Rb2, Rh4, Rg5, Gintonin	• Stimulate Akt/mTOR pathway • Inhibit STAT3 signanling pathway • Decrease Atrogin-1, MuRF-1	[16,17]
2. Anti-inflammatory effect	Rb1, Rb2, Rd, Rg1	• Suppress TNF-α, IL-6 production • Inhibit NF-κB expression via targeting IRAK-1 • Activates Nrf2 signaling pathway	[18,19,20,21]
3. Anti-apoptotic effect	Rb1, ginseng-derived oligopeptides, Compound K	• Decrease NR2B/ERK/CREB/BDNF signaling activation • Decrease rH2AX foci • Ameliorate H2O2-induced neuronal death through activation of Nrf2/HO-1 axis	[22,23,24]
4. Anti-cancer effect	Rh2, Rk1, Rk2, Compound K, 20(S)-Rg3	• Inhibit the proliferation of cancer cells	[25]
5. Anti-aging effect	Rb1, Rb3, Rh2	• Suppression of MAPK signaling pathways • Alleviate DNA damage • Activate DNA repair mechanisms	[22,24,26]

Ginseng is also believed to support cognitive function in older adults. A cohort study involving 6422 elderly Koreans reported that lifelong ginseng consumption was linked to improved cognitive performance in later years [27]. Additionally, ginseng exhibits immunomodulatory effects against bacterial, fungal, and viral infections. An eight-week clinical study in humans demonstrated that daily supplementation with 2 g of Korean red ginseng enhanced immune function by increasing T-cell, B-cell, and white blood cell counts [28]. The primary bioactive compounds in ginseng, ginsenosides, have demonstrated anticancer properties against various malignancies, including breast, brain, liver, gastric, and lung cancers, by reducing inflammation, providing antioxidant protection, and alleviating chemotherapy-induced side effects [29].

Chronic inflammation is a central regulator of muscle-wasting conditions, including cachexia, sarcopenia, and other diseases. Elevated pro-inflammatory cytokines, including TNF-α, IL-1β, and IL-6, activate catabolic pathways such as NF-κB and JAK/STAT3, leading to enhanced ubiquitin-proteasome-mediated protein degradation and suppression of muscle protein synthesis [5]. Ginseng and ginsenosides exhibit potent anti-inflammatory effects through regulating multiple signaling pathways. Accumulating evidence suggests that ginsenosides Rg1, Rb1, Rd, and compound K mitigate inflammatory signaling by inhibiting NF-κB signaling, phosphorylation of MAPKs (p38, JNK, ERK), and reducing the inflammatory cytokines (TNF-α, IL-1β, and IL-6) [30]. In LPS-induced macrophages, ginsenoside Rb1 reduces serum TNF-α and IL-6 levels in a cancer-induced cachexia mouse model. Moreover, Rb1 modulates TLR4/dimerization, thereby downregulating MyD88-dependent activation of NF-κB and MAPK signaling [31]. In addition, ginsenoside Rg3 attenuates inflammation by specifically inhibiting the NLRP3 inflammasome activation, blocking IL-1β production, and caspase-1 activation in macrophages, thereby protecting the mice from endotoxic shock [32]. Taken together, targeting the inflammatory pathway is a critical mechanism through which ginseng exerts protective effects against pathological muscle atrophy. Despite extensive research into the pharmacological properties and therapeutic benefits of ginseng and its bioactive constituents, studies specifically examining its role in muscle-wasting disorders such as cachexia, anorexia, sarcopenia, obesity, and aging remain limited. Therefore, this review seeks to provide a comprehensive analysis of the potential therapeutic effects of ginseng and ginsenosides in muscle-related conditions and disorders through the anti-inflammatory mechanism.

## 2 Structural and chemical characteristics of ginsenosides

**Fig. 1:**
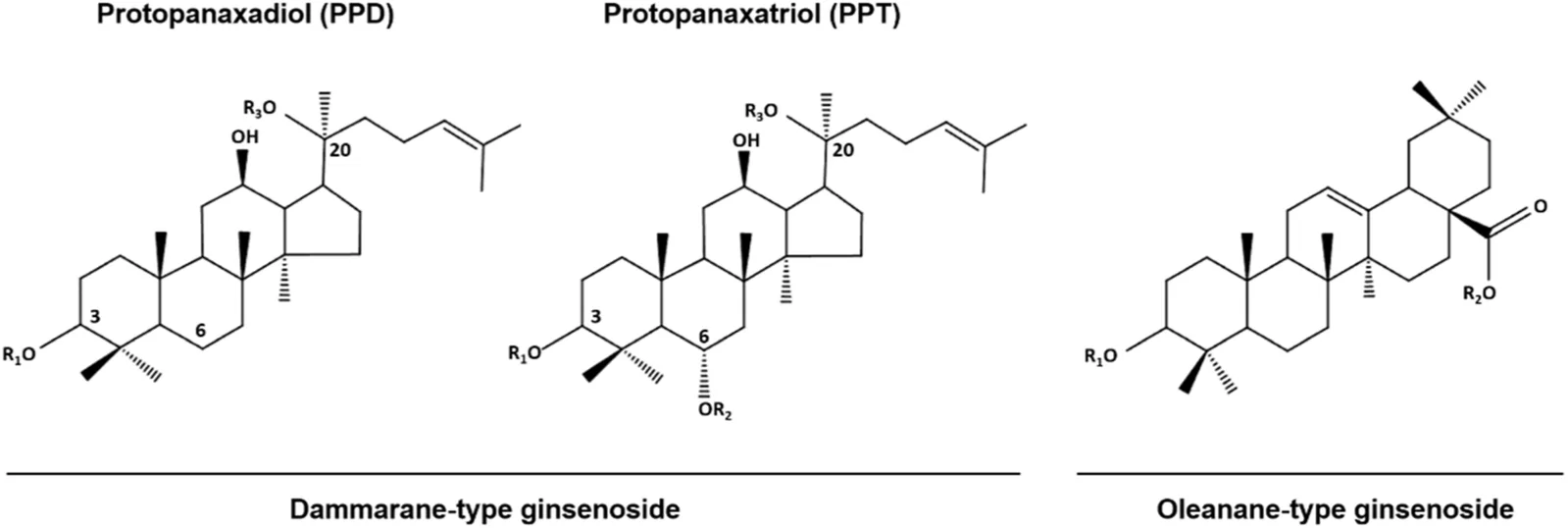
**Chemical structures of the backbone of ginsenoside** Chemical structural classification of ginsenosides. Dammarane-type ginsenosides are further classified into protopanaxadiol-type and protopanaxatriol-type depending on whether a hydroxyl group is present at the C-6 position. Oleanane-type ginsenosides have a pentacyclic moiety.

Ginsenosides are the principal bioactive constituents of *Panax species*. To date, over 200 ginsenosides have been identified. According to TM-MC, an enhanced chemical database of medicinal materials in Northeast Asian traditional medicine, over 100 ginsenosides have been reported in *Panax ginseng* Meyer with supporting evidence (Supplementary Table 1) [33]. Ginsenosides can be separated using thin-layer chromatography. They are commonly named sequentially from low to high retention factor values as ginsenoside ‘Rx’ (x = o, a to h). For example, Ra exhibits the lowest polarity, and Rb is comparatively more polar [34]. Ginsenosides are also classified into dammarane-type and oleanane-type. Dammarane triterpenoids comprise a tetracyclic moiety and side chain moieties [35]. Dammarane-type is further categorized into protopanaxadiol (PPD), protopanaxatriol (PPT), and ocotillol based on their aglycone moiety. PPD-type ginsenosides possess sugar moieties attached to the hydroxyl groups at carbons C-3 and C-20. Similarly, PPT-type ginsenosides form a glycoside with sugar moieties attached to the hydroxyl groups at carbons C-6 and C-20 [36]. Oleanane is a pentacyclic terpenoid (Fig. 1). These classifications are important for understanding the diverse biological activities associated with different ginsenoside structures.

**Fig. 2:**
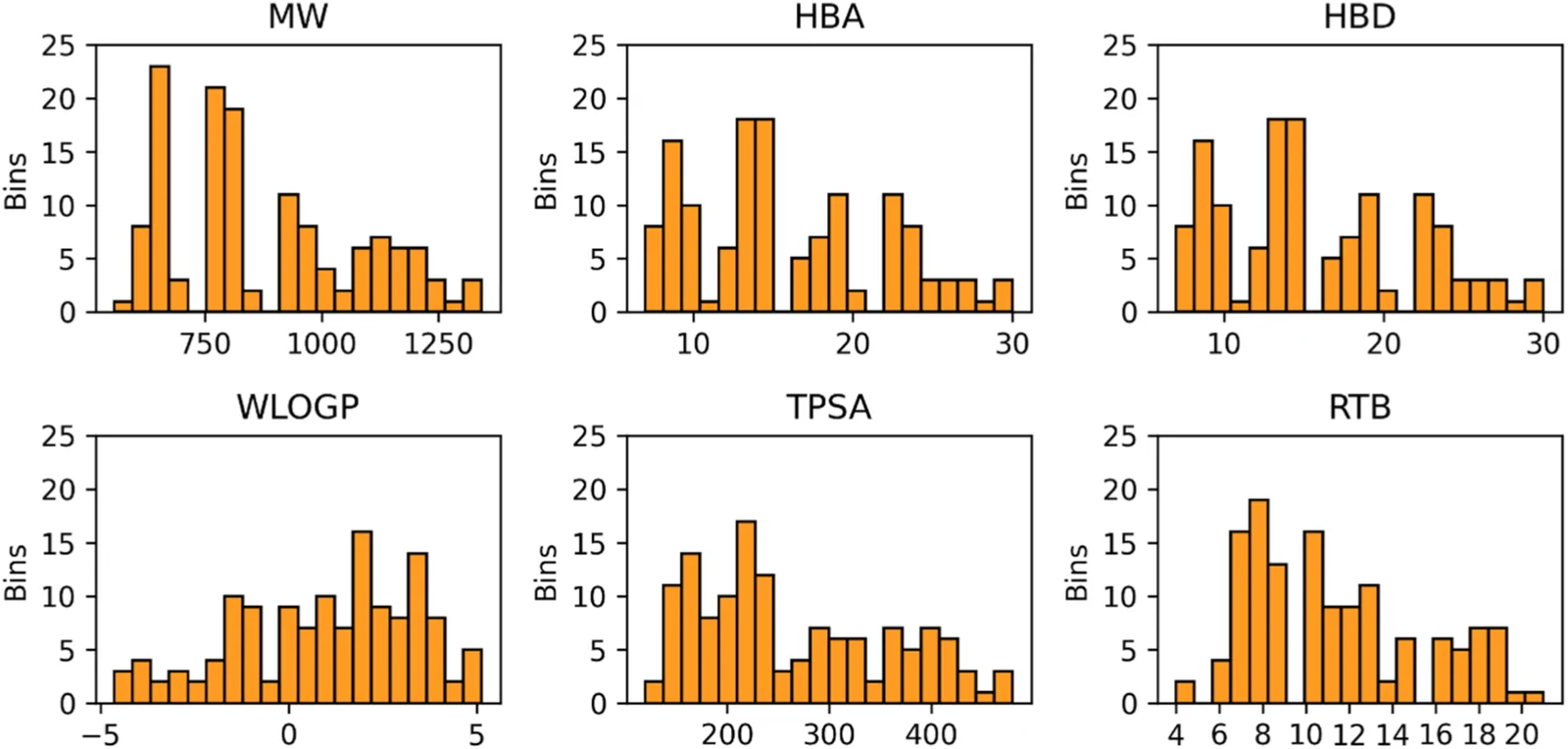
**Distribution of representative physicochemical properties of ginsenosides** Physicochemical properties of ginsenosides are investigated using RDKit software. MW; Molecular weight, HBA; Hydrogen bond donors, HBD; Hydrogen bond acceptors, WLOGP; lipophilicity, TPSA; Topological polar surface area, RTB; Rotatable bonds.

We investigated the distribution of representative physicochemical properties using RDKit, including molecular weight, WLogP, hydrogen bond donors, hydrogen bond acceptors, topological polar surface area, and the number of rotatable bonds (Fig. 2) [37]. These properties are commonly used in the prediction of absorption, distribution, metabolism, and excretion (ADME), and are integral to assessing Lipinski's rule of five [38]. Although the physicochemical profile suggests that the oral bioavailability of ginsenosides is generally below 20%, many ginsenosides exhibit significant pharmacological effects, including anti-inflammation and anti-cancer [39].

Emerging evidence has demonstrated the structure-activity relationship (SAR) of ginsenosides. Biological activity of ginsenosides is influenced by various structural features, including the number or position of glycosylation, stereochemical structure, and the number of hydroxyl groups. Detailed descriptions are provided in Supplementary Table 2. In general, less glycosylation is likely to improve the biological activity of ginsenosides. For example, Kai et al. reported that ginsenoside with a less sugar moiety effectively inhibited the proliferation of HepG2 hepatocarcinoma cells [25]. This implies that the sugar moiety may reduce the hydrophobicity, therefore decreasing the cellular permeability of ginsenoside. Stereochemical configuration comparison studies also emphasize the SAR of ginsenosides on anti-inflammatory activity. Xianwen Ye et al. demonstrated that ginsenoside S-Rg3 suppressed TNF-α release more effectively than R-Rg3, suggesting the impact of stereochemistry on anti-inflammatory effects [40]. Similarly, PPD-type ginsenosides such as Rb1 and Rb3 promote axonal growth and dendritic formation more effectively than PPT or oleanane-type ginsenosides [41]. Zhang et al. showed the neuroprotective effect of ginsenosides against the amyloid beta-induced *Caenorhabditis elegans* model. In this study, 17 ginsenosides were elucidated, and Rc, Rd, 20(S)-Rg3, Compound K, and Rg1 reduced amyloid beta deposits. Interestingly, among the ginsenosides, PPD-type ginsenosides presented much higher activities than PPT-type or oleanane-type ginsenosides [42]. Furthermore, the location of the double bond formation in ginsenosides implies the importance of the structure of ginsenosides. Ginsenosides with a double bond at C20-21, like ginsenoside Rk1, showed higher anti-cancer activity than ginsenosides with a double bond at C20-C22, including ginsenosides Rg5 and Rh4 [25].

Taken together, the therapeutic efficacy of ginsenosides is influenced by several structural features. In general, the number of sugar moieties is negatively correlated with its biological activity. 20(S)-ginsenosides consistently demonstrated more potent pharmacological effects than the 20(R) epimer. Moreover, PPD-type ginsenosides typically exert stronger anticancer, cytoprotective activity than PPT-type ginsenosides. Current evidence on the SAR of ginsenosides provides a structural foundation for the development of further ginseng-based therapeutics against muscle wasting disorders, including sarcopenia and cachexia.

## 3 Effect of ginseng and ginsenosides on muscle-wasting disorders

### 3.1 Bodyweight and cachexia

In general, well-known muscle-wasting conditions include cancer-associated cachexia, aging-related sarcopenia, and anorexia. The most common characteristics of these conditions and inter-diseases relationships are described in Supplementary Table 3. CAC, whether caused by tumor progression or chemotherapy, is commonly associated with a significant decline in body weight, primarily due to the depletion of skeletal muscle mass and fat tissue [[16], [18], [43]]. Multiple studies have explored the potential benefits of ginseng and its active compounds in counteracting this condition. Gintonin (GT), a lysophosphatidic acid receptor ligand derived from ginseng, has been found to alleviate CAC in mice, leading to increases in tumor-free body weight [17]. While mice receiving phosphate-buffered saline (PBS) exhibited a 9.51% reduction in tumor-free body weight relative to a healthy control group, those treated with gintonin-enriched fraction (GEF) exhibited substantial recovery. Additionally, GEF demonstrated efficacy in preserving muscle mass and improving tumor-free body weight in cachexia models [17].

Another ginseng-derived compound, BST204, a purified dry extract containing multiple ginsenosides such as Rh2 and Rg3, has demonstrated protective effects against chemotherapy-induced bodyweight loss [44]. In mice treated with 200 mg/kg of BST204, reductions in tumor-excluded body weight were notably smaller compared to those receiving only 5-fluorouracil (5-FU) chemotherapy, showing declines of −6% vs. −13% on day 7 and -20% vs. −30% on day 11. BST204 also significantly reduced muscle and fat volume loss (−11% vs. −19% for muscle; −56% vs. −91% for fat) compared to the 5-FU group, suggesting its potential to counteract chemotherapy-induced body composition changes [45].

A novel herbal formulation, SGE, composed of ginseng (Ginseng radix alba), *Atractylodis Rhizoma alba*, and Hoelen, was found to have protective effects in CT-26 tumor-bearing mice. Administration of SGE at 10 and 50 mg/kg restored body weight to approximately 95% and 93.3% of baseline by day 20, whereas untreated cachectic mice lost 5.7% of body weight within five days following tumor cell injection. SGE also demonstrated a protective effect against skeletal muscle and adipose tissue loss without inducing weight gain in healthy mice [44].

However, some studies have demonstrated that not all ginseng extracts effectively counteract cachexia. Research investigating water-extracted ginseng, such as GE5, GE50, and ginsenoside Rb1, found no significant improvement in muscle mass, fat tissue volume, or tumor-free body weight in mice with CT26 cancer-induced cachexia [18]. Despite this, GE5, GE50, and Rb1 reduced circulating levels of pro-inflammatory cytokines, including tumor necrosis factor-alpha (TNF-α) and interleukin-6 (IL-6), which play key roles in cachexia progression [18].

Individual ginsenosides have also been comprehensively assessed for their therapeutic potential. Ginsenoside Rg1 has demonstrated muscle-protective effects [17], while ginsenosides Rb1, Rd, Rg3, Rb2, Rh4, and Rg5 have been implicated in stimulating the Akt/mTOR pathway, which supports muscle growth and suppresses atrophy [16]. Additionally, ginsenoside Rb1 has been shown to suppress TNF-α and IL-6 levels [18,16], suggesting a role in the anti-cachectic effects [18]. Ginsenoside Rd, in particular, has been noted for its ability to counteract muscle wasting by inhibiting the STAT3 signaling pathway [16].

Steamed ginseng berry powder (SGBP) has demonstrated efficacy in increasing muscle mass and body weight in aged mice with sarcopenia, a condition closely linked to cachexia [45]. Additionally, SGBP was found to inhibit key molecules involved in sarcopenia and reduce inflammatory cytokine levels [45]. Recent reports related to the therapeutic effect of ginseng and its bioactive compounds on bodyweight regulation are listed in Supplementary Table 4.

In summary, research suggests that ginseng and its ginsenosides may contribute to maintaining or restoring body weight and alleviating cachexia symptoms, primarily by modulating inflammation and preserving muscle mass [[16], [17], [18], [43], [44]]. However, their therapeutic efficacy varies according to the specific compound, dosage, disease model, and treatment duration [18,45]. Further studies are necessary to elucidate the distinct roles of individual ginseng constituents in cachexia management.

### 3.2 Sarcopenia

Sarcopenia is a progressive condition characterized by a decline in skeletal muscle mass and function, primarily affecting the aging population. This age-related muscle degeneration is classified as primary sarcopenia. Additionally, secondary sarcopenia may occur as a result of diverse conditions, including chronic inflammation, neurodegenerative disorders, and metabolic diseases [46]. Other contributing factors include low body weight, smoking, disrupted sleep patterns, and malnutrition [47]. According to the Global Leadership Initiative in Sarcopenia, reductions in both muscle mass and strength form the basis for diagnosis, with associated impairments in physical function, mobility, and increased fall risk [48].

Skeletal muscle is a complex tissue comprising multinucleated myofibers, connective tissue, nerve fibers, and blood vessels. Myofibers contain contractile proteins, including actin and myosin, which facilitate muscle contraction in response to motor neuron stimulation. The neuromuscular junction (NMJ) serves as the interface between motor neurons and myofibers [46]. Aging can compromise NMJ integrity, leading to denervation, which accelerates sarcopenic progression [49]. Notably, sarcopenic individuals exhibit up to a 50% reduction in type II muscle fibers, which are responsible for rapid ATP production and high-force output [50].

Chronic inflammation is a major driver of sarcopenia in older adults. Pro-inflammatory cytokines such as TNF-α, NF-κB, and IL-6 contribute to sarcopenia by promoting muscle protein degradation through distinct signaling cascades. NF-κB activation induces the expression of MuRF1 and Atrogin-1, E3 ubiquitin ligases that mediate proteasomal protein breakdown [51]. Concurrently, Myogenic differentiation 1 (MyoD) and myogenin, which are essential for myoblast differentiation, are suppressed. IL-6 exacerbates muscle atrophy by modulating the ubiquitin pathway via FoxO3a, thereby upregulating Atrogin-1 and MuRF1.

Members of the TGF-β superfamily play a crucial role in sarcopenia pathogenesis. Specifically, growth differentiation factor (GDF)-8 and GDF-15 negatively regulate muscle growth and regeneration when overexpressed. GDF-8, also known as myostatin, inhibits MyoD expression through the SMAD signaling pathway. Myostatin binds to the activin type IIB receptor (ActRIIB) and Alk 4/5, leading to SMAD2/3 phosphorylation. This complex is translocated to the nucleus, where it suppresses MyoD transcription. GDF-15, also termed macrophage inhibitory cytokine-1 (MIC-1), is typically expressed at low levels under normal physiological conditions. However, its expression increases in pathological states such as cancer and chronic inflammation, leading to muscle wasting via TGF-β-activated kinase 1 activation and apoptosis induction [52]. Additionally, elevated GDF-15 expression promotes autophagy through LC3 upregulation [53].

Skeletal muscle is a key metabolic reservoir, regulating ATP storage and resynthesis. Maintaining muscle mass is essential for metabolic homeostasis and protection against chronic conditions, including obesity, diabetes, and cancer. Both sarcopenia and CAC involve progressive muscle loss and functional decline. CAC is driven by inflammatory cytokines, imbalanced protein metabolism, malnutrition, and neural denervation [54,55]. During tumor progression, cytokines such as TNF-α, IL-6, and interferon-gamma activate the STAT-3 and NF-κB signaling pathways, exacerbating muscle atrophy [56].

Ginsenosides possess potent anti-inflammatory properties relevant to sarcopenia management. Specifically, ginsenosides Rb1 and Rb2 suppress TNF-α production in lipopolysaccharide (LPS)-stimulated macrophages (Raw 264.7 cells), a key component of the tumor microenvironment [19]. Ginsenoside Rb1 inhibits NF-κB expression by targeting IRAK-1, a major regulator of inflammation [20]. Similarly, ginsenoside Rg1 reduces inflammation by downregulating TNF-α, IL-1β, and IL-6 in ethanol-administered rat model [20]. Several studies have confirmed that ginsenosides attenuate pro-inflammatory cytokines, which are primary mediators of CAC. In CAC mouse models, Rb1 administration significantly lowered serum TNF-α and IL-6 levels [18].

Another promising ginseng-derived compound, GT, has demonstrated efficacy in mitigating muscle wasting in C2C12 myotubes and cachexia-affected hindlimb muscles. GT exerts its effects by binding to the lysophosphatidic acid (LPA) receptor, activating Gαi2, which protects against mitochondrial ROS damage and suppresses NOX-2-mediated oxidative stress [17]. This interaction reduces TNF-α-induced MuRF-1, Atrogin-1, and Myostatin expression—key markers of muscle atrophy.

Protein synthesis imbalance is central to sarcopenia. The Akt/mTOR signaling pathway is central to skeletal muscle hypertrophy and atrophy prevention [57]. Akt regulates protein synthesis through mTOR, GSK-3β, S6K, and 4E-BP1 while inhibiting protein degradation via Atrogin-1 and MuRF-1 suppression [58]. Modulation of the Akt pathway has been shown to counteract muscle loss in CAC models. Ginsenoside Rg1 has been shown to prevent starvation-induced muscle degradation by modulating Akt signaling [59].

**Fig. 3:**
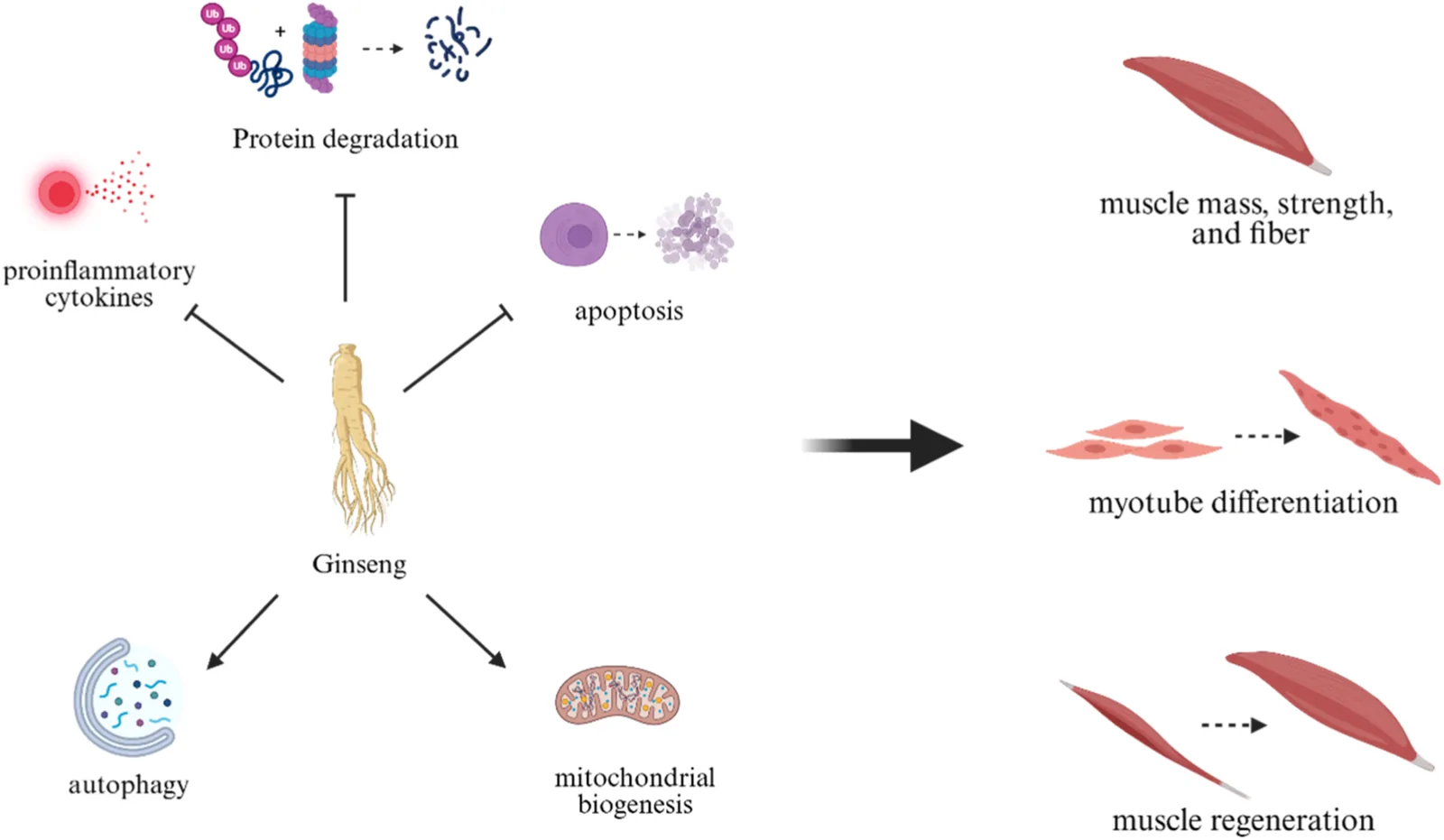
**Ginseng-mediated protective effect against sarcopenia via improvement of the muscle environment** Ginseng has a protective effect on muscle via muscle mass and strength improvement, myotube differentiation, and muscle regeneration. These beneficial effects are achieved by reducing protein degradation, proinflammatory cytokines production, apoptosis, autophagy, and mitochondrial biogenesis.

Several studies have highlighted the potential of ginseng extract in alleviating sarcopenia in both *in vitro* and *in vivo* models (Fig. 3 and Supplementary Table 5). For example, Korean red ginseng (KRG) extract has been shown to enhance muscle function, repair, and growth by modulating multiple biological pathways. In diabetic postmenopausal women, KRG supplementation increased follistatin and sex hormone-binding globulin (SHBG) levels while reducing troponin, a muscle injury marker [60]. These evidences support the therapeutic potential of ginseng in combating sarcopenia and muscle atrophy.

### 3.3 Anorexia

Malnutrition and CAC are significant contributors to sarcopenia [61]. CAC is a metabolic wasting disorder frequently observed in cancer patients. A hallmark of CAC is diminished appetite, leading to the progressive loss of muscle and fat mass, as well as a decline in energy metabolism. These pathophysiological alterations promote cancer progression and reduce the efficacy of anticancer therapies [62]. CAC is associated with hypothalamic dysfunction, which governs appetite regulation, primarily due to increased inflammatory cytokine activity, disrupted energy metabolism, and neuronal apoptosis associated with aging [63]. The hypothalamus serves as a critical regulatory center for appetite and energy homeostasis, with key neuronal components such as pro-opiomelanocortin (POMC) and neuropeptide Y (NPY) playing pivotal roles [64]. Cancer-induced anorexia is mediated, in part, by inflammatory cytokines, such as interleukin-1 beta (IL-1β), secreted by tumor tissues, which downregulate POMC and NPY expression in the hypothalamus. Consequently, therapeutic strategies aimed at restoring POMC and NPY expression have been proposed for alleviating CAC [64]. Research focused on restoring hypothalamic function impaired by cancer is therefore essential for the development of effective interventions against CAC. Recent studies have highlighted the potential of herbal extracts containing ginsenosides, as well as purified ginsenosides, in mitigating CAC symptoms (Supplementary Table 6). Specific ginsenosides, including Rg and Rh, have demonstrated anticancer properties [[65], [66], [67]]. Additionally, these compounds have been recognized for their significant anti-inflammatory effects [68].

Anxiolytic treatments often target hypothalamic structures, such as the paraventricular nucleus (PVN), which is involved in appetite regulation, such as food intake. Some studies suggest that ginsenoside-containing herbal extracts may promote appetite regulation in CAC models, underscoring their therapeutic potential. Studies have reported that traditional Japanese herbal medicine Rikkunshito, which contains ginseng, alleviated cisplatin-induced anorexia and modulated neuronal components, including POMC, CART, NPY, and ARC [[69], [70], [71]]. Furthermore, clinical research demonstrated that Rikkunshito improved appetite loss in lung cancer patients receiving cisplatin treatment [72]. Liujunzi Decoction (LJZD), which is a traditional Chinese formulation containing ginseng, also modulated the JAK-STAT signaling pathway and regulated the expression of hypothalamic anorexigenic and orexigenic neuropeptides [73]. Traditional Korean medicine formulations containing ginseng, such as Yukgunja-tang and Sipjeondaebo-tang, have also demonstrated potential in alleviating CAC-related anorexia [74]. These findings indicate that ginseng alleviates appetite loss in animal models or cancer patients through multiple mechanisms, including modulation of the JAK-STAT pathway and the expression of the neuronal components. Further research is warranted to explore the therapeutic potential of ginsenosides in the treatment of CAC.

Anorexia is a multifaceted condition with diverse underlying causes, particularly among cancer patients and individuals receiving chemotherapy [75]. Ginseng species, including Asian Ginseng, Korean Red Ginseng, and American Ginseng, are widely recognized in Traditional Oriental Medicine for their ability to restore energy, enhance overall health, and reduce fatigue, factors closely associated with anorexia [76]. Recent pharmacological studies have indicated that ginsenosides, the primary bioactive constituents of ginseng, possess anti-inflammatory, antioxidant, immunomodulatory, and anticancer properties, which may contribute to their anorexia-ameliorating effects [74]. Several clinical studies support the therapeutic potential of ginseng for anorexia (Supplementary Table 7). A preliminary study conducted by Yennurajalingam et al. demonstrated that high-dose *P. ginseng* significantly improved appetite in patients experiencing cancer-related fatigue (CRF) [77]. Additionally, a randomized controlled trial in colorectal cancer patients undergoing chemotherapy reported that Korean Red Ginseng supplementation reduced fatigue and improved overall quality of life, including appetite improvement [78]. Specific formulations such as Sipjeondaebo-tang and Yukgunja-tang, both containing ginseng radix, have also been reported to be effective in managing cancer-related anorexia [74,79].

Preclinical studies in animal models provide additional support for ginseng's role in alleviating anorexia caused by chemotherapy (e.g., cisplatin) and immune activation (e.g., LPS) [74,80]. Ginseng radix and specific ginsenosides, such as Rg3, have demonstrated protective effects against anorexia-associated reductions in food intake and body weight [74,80]. The proposed mechanisms of action include suppression of inflammation, modulation of serotonin and cytokines, regulation of appetite-related hormones such as ghrelin and leptin, and the modulation of neuropeptides implicated in appetite control [74]. Additional ginseng-containing formulations, such as Bojungikki-tang, Ban-xia-xie-xin-tang, Dai-kenchu-to, Ninjin'yoeito (NYT), and bukuryoingohangekobokuto (BRGHT), have demonstrated efficacy in enhancing digestive function and mitigating anorexia symptoms [81,82].

Overall, existing evidence underscores the potential of ginseng and its bioactive ginsenosides as promising therapeutic agents for cancer-associated anorexia. Future studies should focus on elucidating the precise molecular mechanisms underlying ginseng's appetite-modulating effects and further validating its clinical efficacy in patients with cancer-associated anorexia.

## 4 Aging and ginseng

Aging is an unavoidable biological process characterized by progressive physiological decline and structural alterations. Historically, *P*. *ginseng* has been valued for its potential to promote longevity [83]. Over the past decades, extensive research has demonstrated its therapeutic efficacy in mitigating age-related diseases, including cancer, metabolic disorders, cardiovascular dysfunction, and neurodegenerative conditions. Zanuso et al. reviewed the role of *P*. *ginseng* in counteracting aging-associated pathologies, such as apoptosis, mitochondrial dysfunction, inflammation, oxidative stress, and metabolic dysregulation. Among its bioactive constituents, ginsenosides have been shown to modulate aging by exerting neuroprotective effects, regulating immune responses, and promoting DNA repair their antioxidant and anti-inflammatory properties [84].

Additionally, volatile oils extracted from ginseng have been reported to extend the lifespan of model organisms, such as *Drosophila melanogaster* and *Caenorhabditis elegans* [85]. Other active constituents of ginseng, including GT and ginsenoside Compound K, have also demonstrated significant anti-aging potential. Ginseng species and their active constituents with reported anti-aging effects are listed in Supplementary Table 8.

**Fig. 4:**
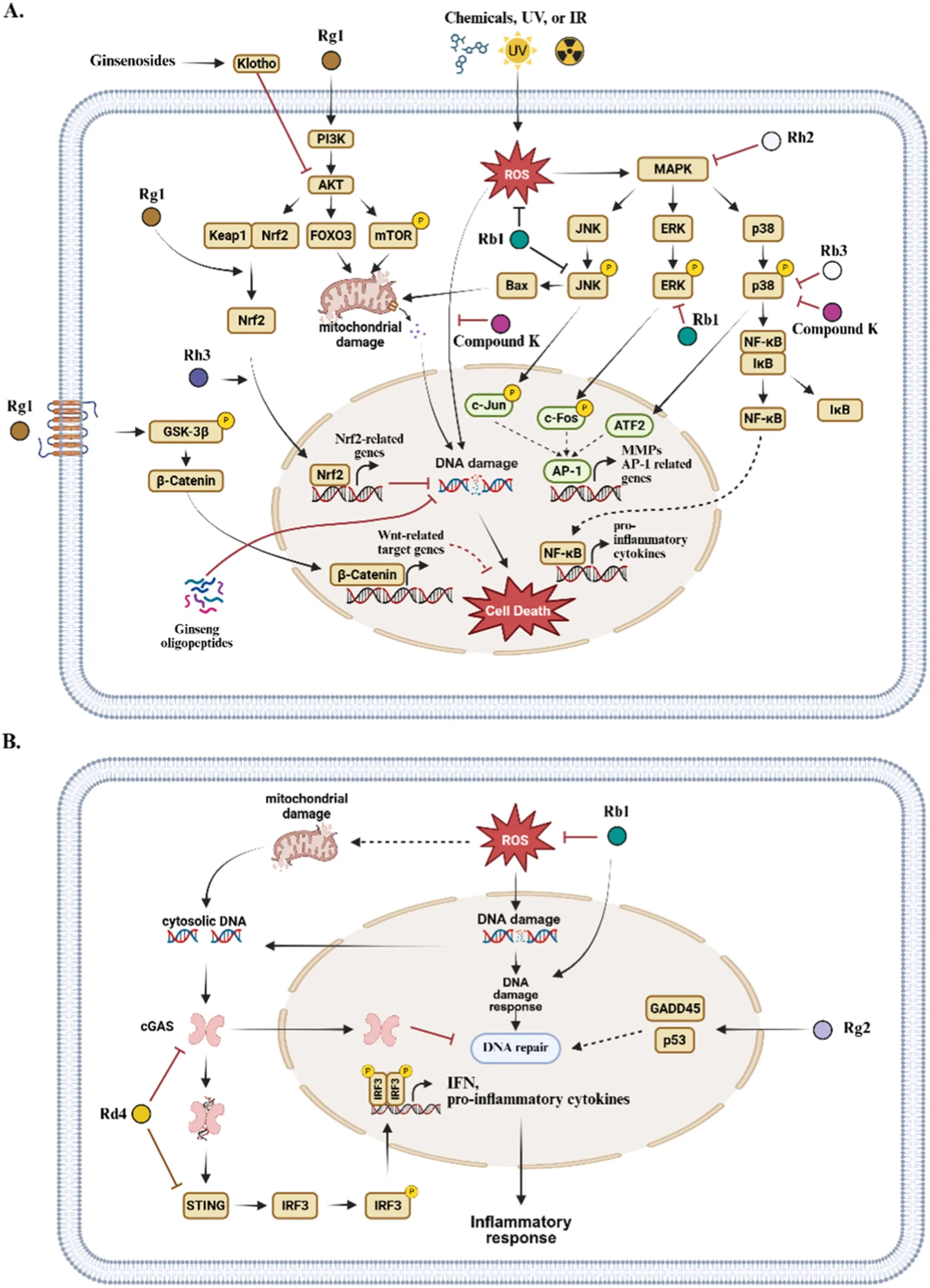
**Anti-aging mechanism of action of ginseng and its active ingredients** (**A**) Protective effect of ginseng against DNA damage. Exogenous stimuli such as UV, irradiation, or chemicals induce ROS leading to DNA fragmentation; ginsenoside Rb1 and compound K mitigate ROS-mediated DNA damage. Ginsenoside Rh2, Rb3, and compound K involved in suppressing the MAPK signaling pathway, affecting DNA damage. Ginsenoside Rg1 protects against DNA damage and cell death by regulation of Nrf2 and Wnt/β-catenin pathway. (**B**) Ginsenosides activate DNA repair mechanism. Ginsenoside Rb1 participates in DNA damage response, consequently contributing DNA repair. Ginsenoside Rd4 is related to the cGAS/STING pathway regulation. When the cGAS/STING pathway is activated, an inflammatory response arises through the IRF3 activation, and nuclear cGAS suppresses DNA repair. Rd4 decreases cGAS and STING expression, thus improving the DNA repair process.

The anti-aging mechanisms of ginseng and its bioactive compounds primarily involve attenuation of endogenous and exogenous oxidative DNA damage and enhancement of DNA damage repair pathways (Fig. 4). Other mechanisms include the exertion of anti-inflammatory effects, promotion of autophagy, modulation of antitumor responses, regulation of gut microbiota composition, and influence on G-protein coupled receptor and lysophosphatidic acid receptor signaling [85]. Ginsenoside Rg1 has been shown to enhance antioxidant defenses by activating the Nrf2 signaling pathway [21]. Exogenous stimuli such as irradiation, UV, or chemicals can induce excessive ROS production, which leads to DNA damage and consequently results in cell death [86]. Specifically, ginsenoside Rb1 has been reported to directly scavenge ROS, which indicates the anti-aging effect of ginsenoside [87]. In addition, ginsenosides exhibited protective effects via the suppression of MAPK signaling pathways. JNK activation increases Bax expression, which induces mitochondrial dysfunction and apoptosis, and simultaneously upregulates inflammatory cytokines and senescence-associated secretory phenotypes [88]. Yang et al. demonstrated that ginsenoside Rb1 attenuates methamphetamine-induced neurotoxicity by reducing ERK and CREB phosphorylation [22]. Lipopolysaccharides are known to induce DNA damage. Ginsenoside Rh2 has been reported to decrease LPS-induced acute lung injury by suppressing pro-inflammatory mediators like iNOS, COX-2, and the activation of MAPK [89]. Collectively, ginsenoside Rh2 may indirectly alleviate oxidative DNA damage. Ginsenoside Rb3 attenuated the phosphorylation of p38 MAPK induced by the cigarette smoke extract, thus preventing fibroblast and epithelial cells from cellular injury through an increase in anti-oxidative activity [90]. Although direct evidence of DNA damage by ginsenoside Rb3 has not yet been reported, p38 MAPK is closely associated with ROS-mediated cellular damage. Consequently, ginsenoside Rb3 may indirectly reduce DNA damage through the inhibition of the p38 MAPK pathway [91]. Moreover, ginsenoside Rg3 has been reported to protect normal human fibroblast cells from N-methyl-N′-nitro-N-nitrosoguanidine-induced DNA damage and apoptosis, while it induces DNA damage in human osteosarcoma cells [92]. Zhu et al. reported that ginseng-derived oligopeptides decreased γH2AX foci levels and apoptosis rates in H_2_O_2_-treated HUVECs and PC-12 cells, respectively [93]. Yet another study reported that ginseng-derived compound K suppressed H_2_O_2_-stimulated DNA damage, apoptosis, and mitochondrial dysfunction through the activation of the Nrf2/HO-1 axis [23].

Several ginsenosides have been implicated in activating DNA repair mechanisms. Ginsenoside Rg2 exhibited a protective effect against UVB-induced DNA damage by increasing the DNA repair mechanism [24]. This process is achieved by regulation of GADD45 and the phosphorylation of p53, which are also associated with muscle weakness [26]. Furthermore, Ginseng-derived peptides reduce the accumulation of damaged DNA in the cytosol, a key trigger for cGAS activation, thereby enhancing nuclear and mitochondrial DNA repair mechanisms. The cGAS-STING pathway, activated by cytosolic DNA, acts as a key mediator of cellular senescence, whereas ginsenoside Rd4 has been reported to decrease expression of cGAS and STING [94]. This may attenuate cGAS-mediated DNA repair suppression and reduce inflammation associated with DNA damage [93]. Ginsenoside Rb1 reduces UVB-induced apoptosis by inducing the DNA repair mechanism via upregulation of the nucleotide excision repair complex [95]. Although various ginsenosides have been reported to exert protective effects against several diseases via DNA damage and repair mechanisms, direct evidence linking these effects specifically to aging and senescence remains limited.

## 5 Ginseng and muscle atrophy-related signaling pathways

### 5.1 HIF-1 signaling

The hypoxia-inducible factor 1 (HIF-1) signaling pathway is a critical therapeutic target in cancer, as it regulates key cellular processes, including glycolysis, lipid metabolism, glycogen synthesis, mitochondrial autophagy, reactive oxygen species (ROS) production, and sarcopenia. Inhibition of HIF-1 activity has been shown to suppress cancer cell survival and proliferation while simultaneously enhancing muscle growth and adipogenesis. Several naturally derived inhibitors of HIF-1 and HIF-2 signaling are currently under clinical evaluation [5]. Recent studies have highlighted the potential of ginseng and its bioactive compounds in modulating the HIF-1 pathway. For instance, Hao et al. reviewed research evidence suggesting that ginseng and its constituents can influence multiple signaling cascades, including calcium, insulin, HIF-1, AGE-RAGE, and cAMP pathways [96]. Traditional Qi-tonifying herbs, such as Ginseng radix and *Astragali Radix*, can synergistically modulate cancer progression by inducing HIF-1 [97]. Furthermore, *P. ginseng* extract has been demonstrated to facilitate neural stem cell differentiation into neuroglobin-producing mature neurons by activating astrocytic HIF-1α, facilitating recovery of retinal and brain functions [98].

Among ginsenosides, Rg3 exhibits notable antitumor activity against various cancer types. Notably, Rg3 suppresses Na^+^/H^+^ exchanger 1 (NHE1) expression by targeting the epidermal growth factor receptor (EGFR), ERK1/2, and HIF-1α signaling pathways, thereby exerting protective effects against hepatocellular carcinoma [99]. Moreover, ginseng-derived glucosyl oleanolate has been shown to suppress cervical cancer cell proliferation through dual inhibition of the PI3K/Akt/HIF-1α signaling pathway within the VEGF/VEGFR autocrine axis while concurrently activating Spry 2 in the VEGF/VEGFR2 paracrine pathway [100]. These findings underscore the potential of HIF-1 inhibitors derived from traditional medicinal sources, such as ginseng, as promising alternative therapeutic agents for managing sarcopenia.

### 5.2 Akt/mTOR signaling

The Akt/mTOR signaling is a key regulator in maintaining skeletal muscle mass by regulating anabolic signals and suppressing proteolytic mechanisms. In cancer cachexia, a catabolic condition, phosphorylation of the Akt cascade leads to the downregulation of mTORC1 and its targets. Thus, protein synthesis is affected, leading to FoxO-dependent transcription of Atrogin-1 and MURF1. This transition toward ubiquitin-proteasome and autophagy-lysosomal degradation accelerates muscle wasting [16]. Recent research reported that ginseng and ginsenosides exhibit a protective effect against muscle atrophy by targeting the Akt/mTOR signaling axis [101]. Ginseng promotes Akt phosphorylation, reactivates the mTORC1 signaling pathway, and suppresses FOXO-mediated muscle-specific E3 ligase activity. Additionally, ginseng diminishes inflammatory cytokines and oxidative stress—two major upstream inhibitors of the Akt/mTOR pathway—thereby safeguarding muscle protein synthesis and preventing myofiber degradation [102]. In summary, these findings underscore ginseng as a promising natural therapeutic option that may attenuate muscle atrophy by influencing the Akt/mTOR signaling pathway.

Another clinically important type of catabolic muscle loss is chemotherapy-induced muscle atrophy. Anticancer agents such as Cisplatin and Fluorouracil directly affect skeletal muscle homeostasis by inhibiting Akt phosphorylation, stimulating mitochondrial dysfunction, and increasing ROS production [103,104]. This results in upregulation of Atrogin-1 and MuRF1 through FoxO activation, increased proteolysis, and decreased protein synthesis [104,105]. Cisplatin or 5-FU treatment in experimental models has been shown to reduce myotube diameter, impair myofiber integrity, and decrease muscle strength [103,106]. Among ginsenosides, Rg1, Rg3, and Rd have been shown to reverse Akt phosphorylation, prevent FoxO nuclear translocation, and downregulate Atrogin-1 and MuRF1 in chemotherapeutic-induced atrophy models [59,107,108]. Moreover, ginseng reduces ROS load, upregulates mitochondrial biogenesis via PGC-1α/SIRT1, prevents amplification of inflammatory signals, and, together, fosters anabolic signaling and decreases proteolytic activity [17,109,110].

### 5.3 FoxO signaling

Forkhead box O (FoxO) transcription factors—primarily FoxO 1 and FoxO3—serve as crucial mediators of skeletal muscle atrophy by transcriptionally enhancing catabolic programs. These include the muscle-specific E3 ubiquitin ligases Atrogin-1/MAFbx and MuRF1, as well as genes involved in autophagy–lysosomal pathways. The nuclear translocation and subsequent activation of FoxOs thus expedite the proteasomal and autophagic degradation of myofibrillar proteins in catabolic states (such as fasting, glucocorticoid exposure, inflammation, and cancer cachexia) [111]. From a physiological perspective, the PI3K/Akt signaling pathway phosphorylates and retains FoxOs within the cytoplasm, thereby inhibiting their transcriptional function; when Akt signaling is diminished, or FoxOs are directly activated, the equilibrium tilts towards protein degradation and a reduction in muscle mass [112]. Thus, targeting FoxO signaling constitutes a logical strategy to prevent or reverse muscle wasting. Ginseng and its bioactive ginsenosides (such as Rg1, Rg3, Rg5, Rc, Rh1, Rd) demonstrate antiatrophic properties by influencing upstream kinase networks and by either directly or indirectly diminishing FoxO activity: treatment with ginsenosides leads to the restoration of Akt phosphorylation, thereby facilitating FoxO phosphorylation and cytosolic sequestration, decreases FoxO3a nuclear localization, and reduces the transcription of Atrogin-1 and MuRF1 in various cell and rodent models of atrophy induced by dexamethasone, disuse, or high glucose [113]. In addition to Akt-dependent inhibition of FoxO, ginseng also diminishes upstream inflammatory and oxidative triggers (NF-κB, STAT3, ROS) that facilitate FoxO activation. Furthermore, it enhances mitochondrial function (PGC-1α/SIRT1), which together leads to a reduction in autophagic flux and ubiquitin-proteasome activity in weakened muscle [16]. Preclinical studies involving cultured myotubes and aged rodents that have been treated with glucocorticoids or are in a cachectic state consistently demonstrate that ginseng preparations or isolated ginsenosides reduce FoxO-driven transcriptional activities, maintain the cross-sectional area of myofibers, and enhance muscle strength—thereby supporting the therapeutic potential of ginseng as an adjunctive approach to combat sarcopenia and cachexia through the modulation of FoxO signaling [114].

## 6 Potential therapeutic effects of ginseng-related compounds in muscle wasting conditions

Ginseng contains several emerging bioactive compounds that are promising for the treatment and prevention of diseases, including cancer cachexia, chemotherapy-induced myopathy, glucocorticoid-induced atrophy, disuse atrophy, and aging-related sarcopenia [17,109,115]. All muscle-wasting diseases share common pathological features, including oxidative stress, mitochondrial dysfunction, chronic inflammation, activation of proteolytic systems, impaired anabolic signaling, and metabolic dysregulation [16,116]. The most notable compounds that ginseng contains are ginsenosides (Rb1, Rg1, Rg3, and compound K), along with polysaccharides and gintonin [59,107,110]. All these compounds differ in composition and target distinct pathways related to muscle wasting, as shown in Table 2 below.

**Table 2:** Comprehensive mechanistic overview of ginseng-related compounds in muscle wasting conditions.

Pathophysiological Domain	Alteration Target	Ginseng-Related Compounds	Molecular Targets/Pathways	Cellular Effects	Functional/Clinical Implication	Relevant Disease Context	References
1. Oxidative Stress	↑ ROS, mitochondrial DNA damage	Rb1, Rg1, Rc	Nrf2 activation; ↑ PGC-1α; ↓ intracellular ROS	Improved redox balance; reduced oxidative injury	Preservation of myofiber integrity	Cancer cachexia; chemotherapy-induced myopathy; sarcopenia	[110,117]
2. Mitochondrial Dysfunction	↓ ATP; impaired biogenesis	Rg1, Rg3, Rc, RGE	↑ PGC-1α; stabilization of mitochondrial membrane	Enhanced bioenergetics; improved resilience	Improved muscle endurance and metabolism	Aging-related sarcopenia; glucocorticoid-induced atrophy; cachexia	[107,110,118]
3. Inflammatory Activation	↑ TNF-α, IL-6; NF-κB activation	Total extract; Rb1, Rd	NF-κB inhibition; cytokine suppression	Reduced inflammatory signaling	Decreased inflammation-driven catabolism	Cancer cachexia; chronic inflammatory disorders	[18,17,102]
4. Ubiquitin–Proteasome System	↑ Atrogin-1, MuRF1 expression	Rb1, Rg1, Rc, Rd	FoxO3a inhibition; suppression of E3 ligases	Reduced proteasomal degradation	Maintenance of muscle protein content	Disuse atrophy; chemotherapy-induced wasting; glucocorticoid-induced atrophy	[59,102,110]
5. Autophagy Dysregulation	Excessive autophagic flux	Rg3, Red Ginseng	AMPK/mTOR modulation; p38 MAPK regulation	Restoration of autophagic homeostasis	Stabilization of muscle proteostasis	Chemotherapy-associated myopathy; metabolic stress-related muscle loss	[107,119,120]
6. Anabolic Suppression	↓ PI3K/Akt/mTOR signaling	Rb1, Rg1, Rc, FRG	Akt phosphorylation; mTOR activation	Enhanced protein synthesis	Promotion of muscle mass retention	Sarcopenia; cancer-associated muscle wasting	[109,121]
7. Apoptosis	↑ Bax; ↑ Caspase-3; ↓ Bcl-2	Rg3, Rg1	↑ Bcl-2/Bax ratio; Caspase-3 inhibition	Reduced myotube cell death	Protection against myofiber loss	Chemotherapy-induced myopathy; advanced cachexia	[59,107,122]
8. Metabolic Stress	Impaired energy balance; ATP loss	Rg3, Gintonin, RGE	AMPK regulation; metabolic enzyme modulation	Improved metabolic flexibility	Support of systemic metabolic stability	Cachexia; metabolic syndrome-associated muscle decline	[17,107,118]
9. Functional Decline	Decreased mass and grip strength	Whole extract; FRG, GBE	Multi-target signaling modulation	Increased fiber CSA; improved grip strength	Improvement in physical performance	Sarcopenia; chronic disease-related muscle weakness	[120,123,124]

## 7 Conclusions

Emerging evidence suggests that ginseng and its bioactive compounds hold significant therapeutic potential for a range of muscle-wasting disorders, cancer-associated metabolic syndromes, and aging-related conditions. The diverse pharmacological effects of ginseng, including its antioxidant, anti-inflammatory, neuroprotective, and metabolic-regulating properties, contribute to its efficacy in mitigating sarcopenia, anorexia, and aging-associated physiological decline. Moreover, the anti-inflammatory potential of ginseng and ginsenosides and their regulatory role on key molecular pathways such as Akt/mTOR, NF-κB, FoxO, and HIF-1 signaling further highlights the significance of ginseng on muscle improvement, appetite regulation, and cancer therapy. Ginsenosides can also improve mitochondrial function, control cytokine expression, and influence neuropeptide activity. These properties suggest their potential as therapeutic agents for preventing and managing muscle wasting disorders and related metabolic dysfunctions. Given the promising preclinical and clinical findings, further research is warranted to elucidate the precise mechanisms of ginseng's bioactive constituents and to explore their integration into conventional therapeutic strategies for inflammation-associated muscle wasting diseases, and other metabolic disorders.

## Conflicts of interest statement

The authors declare no conflict of interest.
