# Supplementary material for: Therapeutic potential of ginseng and ginsenosides on muscle wasting disorders: Cachexia, sarcopenia, anorexia, and aging
Source: J Ginseng Res. 2026 Mar 17;50(5):101021. doi: 10.1016/j.jgr.2026.101021 (PMC13554451; doi:10.1016/j.jgr.2026.101021)
Supplement: Supplementary file 1 [file mmc1.docx]

**Supplementary Table 1. Representative ginsenosides in *Panax ginseng* Meyer, and Korean Red ginseng from the TM-MC database**

| **Name** | **Medicine** | **PubChem ID** | **References** |
| --- | --- | --- | --- |
| 20(22)E-ginsenoside Rg9 | Korean Red ginseng | 102527434 | [1] |
| 20(22)Z-ginsenoside Rh4, Rs4 | Korean Red ginseng | 126612655, 101111172 | [1] |
| 20(E)-ginsenoside F4 | *Panax ginseng* Meyer, Korean Red ginseng | 102179726 | [1, 2] |
| 20(R)-ginsenoside Rf | Korean Red ginseng | 122389843 | [1] |
| 20(R)-ginsenoside Rg2 | *Panax ginseng* Meyer, Korean Red ginseng | 75412551 | [3, 4] |
| 20(R)-ginsenoside Rg3 | *Panax ginseng* Meyer, Korean Red ginseng | 46887680 | [5] |
| 20(R)-ginsenoside Rh1 | *Panax ginseng* Meyer, Korean Red ginseng | 21599923 | [4, 6] |
| 20(R)-ginsenoside Rh1 6'-acetate | Korean Red ginseng | 162854817 | [7] |
| 20(R)-ginsenoside Rh2 | *Panax ginseng* Meyer, Korean Red ginseng | 54580480 | [2, 4] |
| 20(R)-ginsenoside Rs3 | *Panax ginseng* Meyer, Korean Red ginseng | 101253540 | [3, 8] |
| 20(R)-notoginsenoside R2 | *Panax ginseng* Meyer, Korean Red ginseng | 16757287 | [1] |
| 20(S)-ginsenoside Rh1 6'-acetate | *Panax ginseng* Meyer, Korean Red ginseng | 122196265 | [7] |
| 20-gluco-ginsenoside Rf | *Panax ginseng* Meyer, Korean Red ginseng | 24721561 | [1, 4] |
| 23-O-methylginsenoside Rg11 | Korean Red ginseng | 122184438 | [1] |
| 24(R)-ginsenoside Rg7 | *Panax ginseng* Meyer | 101096475 | [3] |
| 25-OH-20(R)-ginsenoside Rh1, Rh2 | *Panax ginseng* Meyer | 21599927, 71517771 | [9] |
| 25-OH-20(S)-ginsenoside Rh1, Rh2 | *Panax ginseng* Meyer | 21599926, 71517770 | [9] |
| 25-OH-ginsenoside Rh4 | *Panax ginseng* Meyer | 102471598 | [10] |
| 3-oxo-20(R)-ginsenoside Rh1 | Korean Red ginseng | 145966465 | [7] |
| 6'-O-acetyl-20(S)-ginsenoside Rh2 | *Panax ginseng* Meyer | 101253541 | [9] |
| acetyl ginsenoside F1 | *Panax ginseng* Meyer | 154809462 | [11] |
| acetyl ginsenoside Rd, Rg1 | *Panax ginseng* Meyer, Korean Red ginseng | 21637588, 11136628 | [12] |
| acetyl ginsenoside Rb1 | *Panax ginseng* Meyer | 101679657 | [12] |
| acetyl ginsenoside Re | *Panax ginseng* Meyer | 101253546 | [6] |
| floral ginsenoside M, N, P | *Panax ginseng* Meyer | 101458199, 101458200, 163089508 | [6] |
| floral ginsenoside E, F | *Panax ginseng* Meyer | 16655212, 16655213 | [12] |
| ginsenoside F1, F2, F3 | *Panax ginseng* Meyer, Korean Red ginseng | 9809542, 9918692, 46887678 | [8, 12] |
| ginsenoside F4 | *Panax ginseng* Meyer, Korean Red ginseng | 102004835 | [4, 13] |
| ginsenoside F5 | *Panax ginseng* Meyer | 46887590 | [12] |
| ginsenoside Ia | *Panax ginseng* Meyer | 118721996 | [2] |
| ginsenoside Ki, Km, La | *Panax ginseng* Meyer | 44607221, 44607222, 163030965 | [12] |
| ginsenoside M7cd | *Panax ginseng* Meyer | 101596945 | [3] |
| ginsenoside Mc | *Panax ginseng* Meyer | 9896928 | [13] |
| ginsenoside Ra0 | *Panax ginseng* Meyer | 102601548 | [14] |
| ginsenoside Ra1, Ra2, Ra3 | *Panax ginseng* Meyer, Korean Red ginseng | 100941542, 100941543, 100941544 | [5] |
| ginsenoside Ra6 | *Panax ginseng* Meyer | 118721997 | [6] |
| ginsenoside Rb1, Rb2, Rb3, Rc, Rd | *Panax ginseng* Meyer, Korean Red ginseng | 9898279, 6917976, 12912363, 12855889, 11679800 | [5, 12] |
| ginsenoside Rd2 | *Panax ginseng* Meyer | 21672569 | [2] |
| ginsenoside Re, Re1, Re2, Re3 | *Panax ginseng* Meyer, Korean Red ginseng | 441921, 122397102, 101717751, 122397101 | [5, 12] |
| ginsenoside Re4, Re5 | *Panax ginseng* Meyer | 102197694, 52951889 | [12] |
| ginsenoside Re7 | *Panax ginseng* Meyer | 132523928 | [15] |
| ginsenoside Rf | *Panax ginseng* Meyer, Korean Red ginseng | 441922 | [5, 12] |
| ginsenoside Rf-1a | *Panax ginseng* Meyer, Korean Red ginseng | 122184437 | [12] |
| ginsenoside Rf2 | Korean Red ginseng | 129010973 | [4] |
| ginsenoside Rg1 | *Panax ginseng* Meyer, Korean Red ginseng | 441923 | [5, 12] |
| ginsenoside Rg10 | Korean Red ginseng | 102527435 | [16] |
| ginsenoside Rg2 | *Panax ginseng* Meyer, Korean Red ginseng | 21599924 | [5, 12] |
| ginsenoside Rg3 | *Panax ginseng* Meyer, Korean Red ginseng | 9918693 | [4, 12] |
| ginsenoside Rg5 | *Panax ginseng* Meyer, Korean Red ginseng | 11499199 | [5] |
| ginsenoside Rg6 | *Panax ginseng* Meyer, Korean Red ginseng | 91895489 | [3, 4] |
| ginsenoside Rg7 | *Panax ginseng* Meyer | 13992214 | [2] |
| ginsenoside Rg8 | Korean Red ginseng | 102179725 | [8] |
| ginsenoside Rh1 | *Panax ginseng* Meyer, Korean Red ginseng | 12855920 | [4, 12] |
| ginsenoside Rh11 | *Panax ginseng* Meyer | 46224641 | [2] |
| ginsenoside Rh16 | *Panax ginseng* Meyer, Korean Red ginseng | 122196266 | [2] |
| ginsenoside Rh19 | *Panax ginseng* Meyer | 145975377 | [12] |
| ginsenoside Rh2 | *Panax ginseng* Meyer, Korean Red ginseng | 119307 | [4, 12] |
| ginsenoside Rh3 | *Panax ginseng* Meyer, Korean Red ginseng | 122196267 | [4, 8] |
| ginsenoside Rh4 | *Panax ginseng* Meyer, Korean Red ginseng | 21599928 | [3, 4] |
| ginsenoside Rh5, Rh6, Rh7, Rh8 | *Panax ginseng* Meyer | 10699455, 101096471, 101096472, 101096473 | [3] |
| ginsenoside Rh9 | *Panax ginseng* Meyer | 163044038 | [3] |
| ginsenoside Rk2 | *Panax ginseng* Meyer, Korean Red ginseng | 138548936 | [4, 8] |
| ginsenoside Rk3 | *Panax ginseng* Meyer, Korean Red ginseng | 75412555 | [3, 17] |
| ginsenoside Ro | *Panax ginseng* Meyer, Korean Red ginseng | 11815492 | [5, 12] |
| ginsenoside Ro methyl ester | *Panax ginseng* Meyer, Korean Red ginseng | 101669622 | [12] |
| ginsenoside ROA | *Panax ginseng* Meyer | 101532859 | [3] |
| ginsenoside Rs1. Rs2 | *Panax ginseng* Meyer, Korean Red ginseng | 101596947, 101596948 | [5, 12] |
| ginsenoside Rs3, Rs4 | *Panax ginseng* Meyer, Korean Red ginseng | 100937823, 163020413 | [4, 8] |
| ginsenoside Rs5 | *Panax ginseng* Meyer, Korean Red ginseng | 102021585 | [8] |
| ginsenoside Rs6 | *Panax ginseng* Meyer | 162886991 | [3] |
| ginsenoside Rs7 | *Panax ginseng* Meyer | 101111174 | [3] |
| ginsenoside St2 | *Panax ginseng* Meyer | 162999947 | [12] |
| ginsenoside-Ro-6'-butyl ester | Korean Red ginseng | 122184439 | [1] |
| koryoginsenoside R1, R2 | *Panax ginseng* Meyer | 101682243, 101682244 | [4] |
| malonyl floralginsenoside Rb1, Rb2 | *Panax ginseng* Meyer | 132576324, 132576325 | [12] |
| malonyl floralginsenoside Rc2 | *Panax ginseng* Meyer | 132576328 | [18] |
| malonyl floralginsenoside Rd6 | *Panax ginseng* Meyer | 132576327 | [12] |
| malonyl ginsenoside Ra3 | *Panax ginseng* Meyer, Korean Red ginseng | 163016420 | [12] |
| malonyl ginsenoside Rb1, Rb2, Rc, Rd | *Panax ginseng* Meyer, Korean Red ginseng | 101835367, 14162969, 101835368, 14162967 | [5, 12] |
| malonyl ginsenoside Rb3 | *Panax ginseng* Meyer, Korean Red ginseng | 132554564 | [8, 12] |
| malonyl ginsenoside Re | *Panax ginseng* Meyer, Korean Red ginseng | 132576321 | [8, 12] |
| notoginsenoside A, Fc | *Panax ginseng* Meyer | 6451129, 75412556 | [19] |
| notoginsenoside Fa | *Panax ginseng* Meyer, Korean Red ginseng | 44592853 | [20] |
| notoginsenoside Fd | *Panax ginseng* Meyer | 46887681 | [21] |
| notoginsenoside Fe | *Panax ginseng* Meyer | 90657714 | [12] |
| notoginsenoside Ft1 | *Panax ginseng* Meyer, Korean Red ginseng | 53348538 | [20] |
| notoginsenoside K | *Panax ginseng* Meyer, Korean Red ginseng | 10605887 | [5] |
| notoginsenoside Q | *Panax ginseng* Meyer | 21674164 | [22] |
| notoginsenoside R1, R2, R4 | *Panax ginseng* Meyer, Korean Red ginseng | 441934, 21599925, 44592854 | [5, 12] |
| notoginsenoside R10 | *Panax ginseng* Meyer | 154497114 | [2] |
| notoginsenoside R3 | Korean Red ginseng | 73351440 | [16] |
| notoginsenoside S | *Panax ginseng* Meyer | 21674165 | [22] |
| notoginsenoside T5 | Korean Red ginseng | 102004610 | [16] |
| pseudoginsenoside F11 | *Panax ginseng* Meyer | 21633072 | [23] |
| pseudoginsenoside Rt1 | *Panax ginseng* Meyer | 21633069 | [24] |
| trans-but-2-enoyl-ginsenoside Rd | *Panax ginseng* Meyer | 101945294 | [19] |
| vinaginsenoside R4 | *Panax ginseng* Meyer | 15940177 | [12] |

**Supplementary Table 2. Overview of structural features associated with the biological activity of ginsenosides**

| **Structural features** | **Associated activity** | **References** |
| --- | --- | --- |
| Decrease in glycosylation number | • Ginsenoside with fewer sugar moiety increased binding affinity for glucocorticoid receptor  • PPD or PPT with single sugar moiety through the inhibition of aldose reductase activity  • Ginsenosides with a single C-20 hydroxyl group and one sugar attached to C-3 or C-6 demonstrated more potent RLAR inhibition compared to ginsenosides containing multiple sugar at C-3 or C-6. | [25, 26] |
| Position of glycosylation position (C-3/C-6/C-20) | • C-3 glycosylation yields the highest cytotoxicity against HepG2 cancer cell, followed by C-6 and C-20 | [27] |
| C-20 stereochemistry | • 20(S)-ginsenoside Rg3 inhibited NaV1.2 channel stronger than 20(R)-ginsenoside Rg3  • 20(S)-ginsenoside Rg3 strongly inhibited hydroxyl radical than 20(R)-ginsenoside  • 20(S)-ginsenoside Rh2 has antitumor effect at lower concentration, but 20(R)-ginsenoside Rh2 exhibited weakly inhibitory effect | [28–30] |
| Skeleton type | • PPD-type ginsenoside showed the anticancer activity in comparison to PPT-type ginsenoside  • Ginsenoside with a PPD-type exhibited a greater anti-apoptotic effect under hypoxia/reoxygenation than PPT-type | [31, 32] |
| Position of double bond | • Ginsenoside with a double bond at C-20 to C-21 showed greater antitumor activity those of C-20 to C-22 | [27] |

**Supplementary Table 3. Common mechanisms and inter-diseases relationships between cachexia, sarcopenia, and anorexia**

| Characteristics | Cachexia | Sarcopenia | Anorexia | Key references |
| --- | --- | --- | --- | --- |
| Clinical Definition | Involuntary loss of muscle (±fat) driven by underlying disease (e.g. cancer) | Age-related progressive loss of muscle strength and function | Disease-related (eg. cancer) and/or age-related loss of appetite and/or reduced food intake | [33] |
| Major upstream drivers | Tumor/host inflammation, tumor-derived factors, metabolic dysregulation, and treatment toxicity. | Inflammaging, reduced anabolic signaling, physical inactivity, comorbidities, nutritional insufficiency. | Hypothalamic signals, cytokine-induced appetite suppression, altered gut signals. | [34] |
| Pathways causing muscle loss | Mitochondrial dysfunction, myostatin/activin signaling, autophagy-lysosome, impaired AKT/mTOR, and activation of ubiquitin-proteasome system (MuRF1, Atrogin-1) | Reduced anabolic signaling, increased oxidative stress, mitochondrial dysfunction, neuromuscular junction changes, satellite cell decline. | Indirectly causes effector activation via energy deficit and increases catabolic signaling; central neuropeptide changes (↓NPY, ↑POMC) worsen intake | [35] |
| Role of immunity and inflammation mediators | Central: tumor and host cytokines (IL-6, TNF-α, IL-1β), acute-phase response, systemic metabolic reprogramming. | Inflammaging contributes to anabolic resistance and muscle catabolism. | Cytokines act on hypothalamus to suppress appetite; inflammatory mediators (eg. IL-6) are implicated in anorexia-cachexia. | [33] |
| Metabolic / endocrine changes | Increased resting energy expenditure, adipose tissue browning, insulin resistance, altered lipid and amino-acid metabolism. | Anabolic hormone declines (testosterone, IGF-1), insulin resistance, altered adipokines. | Altered ghrelin, leptin signaling and CRF/corticosteroid changes influence appetite and energy balance. | [36] |
| Nutritional and behavioural contributors | Reduced intake (anorexia) worsens cachexia; systemic inflammation makes feeding insufficient to reverse loss. | Inadequate protein/energy intake and inactivity accelerate sarcopenia; anabolic resistance reduces response to nutrition. | Directly reduces nutrient intake; in disease states interacts with treatment, mood, and sensory changes. | [37] |
| Practical implications for treatment strategy | Multimodal: treat underlying disease, anti-inflammatory/metabolic agents, nutrition, appetite stimulants, exercise where possible | Emphasize resistance exercise, adequate protein/energy, correctable hormone deficits; address comorbidities. | Treat underlying cause (eg. depression, meds), use appetite stimulants when appropriate; address central and peripheral drivers. | [38] |
| Neural control of muscle metabolism | Hypothalamic inflammation increases sympathetic outflow, raising lipolysis and proteolysis (UPS, autophagy activation). | Motor neuron degeneration and impaired neuromuscular junctions reduce muscle activation, accelerating atrophy. | Malnutrition and inactivity impair neuromuscular performance, reducing muscle use and promoting wasting. | [33] |

**Supplementary Table 4. Role of ginseng and its ingredients on bodyweight improvement in cachexia mice model.**

| **Animal model** | **Cachexia Model** | **Experimental Groups** | **Outcome measures on body weight** | **References** |
| --- | --- | --- | --- | --- |
| C57BL/6 mice | Lewis lung carcinoma (LLC1)-induced CAC | GEF (Gintonin-enriched fraction) group  and Healthy PBS-treated group | Improved tumor-free body weight increased the weight of muscles (Soleus, EDL, Gastrocnemius, Tibialis anterior), enhanced muscle strength (grip strength, hanging time), increased cross-sectional area of muscle fibers.  Other index: Reduced levels of Myostatin, MuRF-1, and Atrogin-1 in Gastrocnemius muscle. | [39] |
| BALB/c mice | 5-fluorouracil (5-FU) induced tumor-bearing mice | Untreated nontumor-bearing (NTB) group, untreated tumor-bearing (TB) group, 5-FU treated group (5-FU), 5-FU treated with 100 mg/kg BST204 group (BST204100), and 5-FU  treated with 200 mg/kg BST204 group (BST204200). | Significantly mitigated the decrease in tumor-excluded BW, reduced loss of muscle and fat volume.  Improved inflammation markers (IL-6, histamine, kynurenine), reduced oxidative stress (methionine sulfoxide), improved protein degradation/stabilization ratio, improved glucose-mediated energy production, improved glucose-mediated biosynthesis. | [40] |
| BALB/c mice | C-26 colon cancer-induced cachexia | WEG (Water Extract of Ginseng) group; GE5 (Ginseng Extract 5 mg/kg) group; GE50 (Ginseng Extract 50 mg/kg) group; Rb1 (Ginsenoside Rb1 10.72 mg/kg) group  Healthy control group with PBS; Tumor-bearing control group with PBS (cachexia model) | No significant improvement in tumor-free body weight, Gastrocnemius muscle weight, or fat weight  Significantly reduced TNF-α and IL-6 levels in GE5, GE50, and Rb1 groups. No significant changes in food intake or tumor volume. | [41] |
| BALB/c mice | CT-26 colon adenocarcinoma-induced cachexia | SGE (10 mg/kg and 50 mg/kg) groups - containing ginseng radix alba  Healthy control group with saline; Tumor-bearing control group with saline (cachexia model) | Attenuated weight loss, prevented loss of muscle and adipose tissue, increased body weight in tumor-bearing mice,  No effect on food intake.  Significantly reduced serum IL-6 levels. | [42] |
| C57BL/6 mice | Sarcopenia  (aged mouse model) | SGBP (Steamed Ginseng Berry Powder - low and high doses) group; NSGBP (Non-Steamed Ginseng Berry Powder) group; RGX365 group  Young control group; Aged control group without treatment | Increased total muscle mass per unit of body weight and overall body weight. Increased cross-sectional area of muscle fibers. Enhanced muscle strength.  Reduced mRNA expression of sarcopenia-inducing molecules (atrogin-1, MuRF1). Reduced levels of inflammatory transcription factors and cytokines (IL-1β, IL-6). Increased expression of MyoD and MyoG. Increased expression of MyHC isoforms. | [43] |

**Supplementary Table 5. Role of ginseng bioactive compounds in ameliorating sarcopenia in preclinical studies**

| **Bioactive compound** | **Upregulation** | **Downregulation** | **Model** | **References** |
| --- | --- | --- | --- | --- |
| Korean Red Ginseng | muscle fiber, myogenin | p53, Bax, cytochrome c, caspase 3, p-Akt/mTOR | sciatic nerve dissection in C5BL/6 mouse for inducing muscle atrophy. | [44] |
| Ginsenoside Rg5 | p-38MAPK, MyoD, p-Akt/mTOR, FoxO3a, myotube diameter | Troponin, Atrogin-1 and MuRF-1 | C2C12 | [45] |
| Korean Red Ginseng  (non-saponin fractions) | muscle mass, fiber size, and strength | Atrogin-1, MuRF-1, Pax7, p57, Intracellular lipid accumulation, proliferation | C2C12 and C5BL/6 mouse | [46] |
| Korean Red Ginseng  (non-saponin fractions) | muscle mass, fiber size, and strength, MyoD, MEF-2, myogenin, CD11b+F4/80+ macrophages | Fbx32, MuRF-1, myostatin, IL-1, IL-6, TNF-alpha, CRP. | C5BL/6 mouse (3-6 month young and 20-24 month old mouse) | [47] |
| Gintonin from Korean White Ginseng | Lipolysis, myogenic differentiation, muscle mass, and strength | Body mass, adipocyte size, mitochondrial biogenesis, myogenic differentiation | ICR mouse with high fat diet | [48] |
| Ginsenoside Rb2 | MHC, MyoD, Akt/mTOR signaling, myogenin |  | C2C12, rhabdomyosarcoma cells | [49] |
| Steamed Ginseng Berry Extract (containing PPT- and PPD-type rare ginsenosides (20(S)-Rg2, 20(S)-Rh1, 20(R)-Rg2, 20(R)-Rh1, Rg6, Rg4, Rh4, 20(S)-Rg3, 20(R)-Rg3, Rk1, and Rg5) | Muscle mass, strength, and fiber, MyHC (embryonic, I, IIa, IIb, IIx), MyoD, MyoG | NF-KB, IL-6, IL-1-Beta, Atrogin-1, MuRF-1 | C57BL/6 | [43] |

**Supplementary Table 6. Efficacy of ginseng-related herbal formulations on Anorexia**

| **Name** | **Component** | **Biological activity** | **References** |
| --- | --- | --- | --- |
| Herbal Extract | Ginsenoside Rg1 | Food intake | [50–53] |
| Herbal extract | *Panax ginseng* | Food intake | [54] |
| Liujunzi Decoction (LJZD) | *Panax ginseng* | Food intake | [55, 56] |
| Ninjin-yoeito | Ginsenoside Rg1 | Food intake | [57] |
| Yukgunja-tang | Ginseng radix | Anorexia | [58] |
| Sipjeondaebo-tang | Ginseng radix | Anorexia, food intake, appetite hormone | [59, 60] |
| Bojungikgi-tang | Ginseng radix | Anorexia | [61] |

**Supplementary Table 7. In vitro, in vivo, and clinical studies related to the effect of ginseng on anorexia**

| **In vivo/in vitro studies/**  **Clinical research** | **Experimental Groups** | **Biological effect** | **Assessment Measures** | **References** |
| --- | --- | --- | --- | --- |
| Mice (12 analyzed studies) | Various groups of mice treated with single herbal medicine extracts or mixtures, with ginseng radix.  Control groups: Mice groups treated with cisplatin only or healthy control groups. | Cisplatin-induced anorexia | Body weight: Changes were noted in some studies.  Food intake: Measured in all studies.  Biochemical analysis: Serotonin and its receptors, cytokines (IL-6, TNF-α, IL-1β, IFN-γ, IL-2, IL-4), WBCs (total count, neutrophils, lymphocytes, monocytes, NK cell activity, macrophage phagocytic activity), ghrelin, leptin. | [62] |
| Female ICR mice (n=10) | Rg3 group (20 and 40 mg/kg), Minocycline group (50 mg/kg), Control group (no LPS, no treatment), Model group (LPS 0.83 mg/kg) | LPS-induced depressive-like behavior | Body weight: Reduced by LPS, Rg3 and Minocycline showed protective effects.  Food intake: Reduced by LPS, Rg3 and Minocycline showed protective effects.  Biochemical analysis: Hippocampal KYN, TRP, 5-HIAA, and 5-HT concentrations and KYN/TRP, 5-HIAA/5-HT ratios; hippocampal mRNA of IL-1β, IL-6, TNF-α, IDO; hippocampal protein expression of NF-κB pathway factors; plasma IL-6, TNF-α concentrations; plasma IDO activity. | [63] |
| Cancer patients (n=30, 24 evaluable) | High-dose Asian Ginseng (*P. ginseng*) (800 mg orally daily for 29 days).  No placebo control group in this preliminary study. | Cancer-related fatigue (CRF) | Body weight: Not directly mentioned.  Food intake: Appetite improved (assessed by ESAS). Biochemical analysis: Not performed in this study. | [64] |
| Review (multiple animal studies) | Animal studies using ginseng extracts (Korean ginseng whole extract, black ginseng ethanol extract, Chinese ginseng extract) and ginsenosides (Rb1, Rg1, Rg3, Rh2, compound K).  Obese control groups (e.g., high-fat diet). | Obesity and regulation of appetite-related neuropeptides (anorexia mentioned) | Body weight: Reduction in body weight and fat mass reported in multiple studies.  Food intake: Suppressed orexigenic neuropeptides and promoted anorexigenic neuropeptides (NPY, AgRP decreased; CCK, POMC increased).  Biochemical analysis: Activation of AMPK pathway, reduction in lipid accumulation, decrease in pancreatic lipase activity; correlation between ginseng constituents (campesterol, beta-elemene, ginsenoside Rb1, biotin, pantothenic acid) and neuropeptide genes (ADIPOQ, NAMPT, UBL5, NUCB2, LEP, CCK, GAST, IGF1, RLN1, PENK, PDYN, POMC). | [65] |
| Advanced esophageal cancer patient (case report) | Kampo medicines bukuryoingohangekobokuto (BRGHT) and ninjin'yoeito (NYT). Ginseng is a component in both formulas.  No formal control group (case report). | Anorexia and esophageal tightness | Body weight: Improved nutritional status (indirectly).  Food intake: Recovery of appetite (based on report).  Biochemical analysis: BRGHT reported to modulate cerebral levels of 5-hydroxytryptamine, noradrenaline, and dopamine (based on other cited studies). NYT activates NPY neurons. | [66] |
| Review (multiple human and animal studies) | Review mentions the use of Ginseng and other TCM formulas (Shi-quan-da-bu-tang (TJ-48), Bu-zhong-yi-qi-tang (TJ-41), Huang-qin-tang (PHY906)) as well as acupuncture.  Conventional cancer therapies (chemotherapy, radiotherapy, targeted therapy, immunotherapy). | Positive role of Traditional Chinese Medicine as an adjunctive therapy for cancer (anorexia mentioned) | Body weight: Improved (indirectly through improved QOL and reduced side effects).  Food intake: Reduced anorexia and improved QOL.  Biochemical analysis: Ginsenosides possess anti-inflammatory, antioxidant, immunomodulatory, and anticancer properties (based on cited studies). | [67] |

**Supplementary Table 8. Anti-aging effects of ginseng extract and its bioactive compounds**

| **Extract/Compound** | **Cells/in vivo model** | **Mechanism of action** | **Experiments** | **References** |
| --- | --- | --- | --- | --- |
| saponins from *Panax Japonicus* | RAW264.7 macrophage cells | Anti-aging via iron metabolism | SA-β-gal, p53, p21, γ-H2AX, Cd68, Cd11c, IL-1β, TFR, HO-1, FTH, FTL | [68] |
| Fresh Korean ginseng root extract | Normal human dermal fibroblasts (NHDFs) | Anti-skin aging,  Anti-inflammatory | ROS, MMP-1, IL-1β, IL-6, LOR, AQP3, FLG, and KRT1 | [69] |
| Non-saponin fraction of red ginseng | skeletal muscle cell line (C2C12) | Prevention of aging-related muscle atrophy | Oil red, SA-β-gal, atrogin-1, MuRF-1, Pax7, p21, p57 | [46] |
| Ginsenosides aqueous extract | *Caenorhabditis elegans* | Anti-oxidative and longevity | ROS, NAD+, SIRT1, NRF2 | [70] |
| Korean Red ginseng extract | Human umbilical vein endothelial cells (HUVECs) | Anti-inflammatory and Anti-oxidant | SA-β-gal, ROS NO, NF-κB, p53, p21 | [71] |
| Korean Red Ginseng extract | AGS human gastric epithelial cells | Anti-oxidant, Anti-apoptotic effect, inhibition of oxidative DNA damage | ROS, Bax/Bcl-2, caspase-3, ATM, Mdm2, ARF | [72] |
| Ginseng-derived rhamnogalacturonan I (RG-I) | *C. elegans* | Longevity | ROS, Nrf2, DAF-16 | [73] |
| Red Ginseng extract | C57BL/6 mice (21 months old) | Anti-inflammatory effect  and regulation of antioxidant  enzyme activity | NOS, COX, TNF-α, IL-1β | [74] |
| Oligopeptides from Ginseng | Embryonic NIH/3T3  fibroblasts | Longevity, Anti-senescence, Anti-oxidative, Anti-inflammatory effects | γ-H2AX, ROS, GSH-Px, SOD, MDA | [75] |
| Ginseng volatile oil | *C. elegans* | Increase life span and health span | SOD, MDA | [70] |
| Black ginseng (BG) extract | C57BL/6J mice (18-month-old) | Senolytic activity (Inhibition of p53-dependent p16, p21) | SA-β gal, p53 | [76] |
| Red ginseng | 36-days-old female  *Drosophila*  *melanogaster* | Life-prolonging and anti-aging | Raf1, ERK, p-ERK, AKT, p-AKT | [77] |
| Korean Red ginseng | The House Ear Institute-Organ of Corti 1 (HEI-OC1) cells,  Dec 2−/− (DEC KO) mice | Anti-aging | Dec1, Dec2, Dec25, Il1β, Fabp2 | [78] |
| Compound K | Primary normal human keratinocytes | Anti-skin aging (Inhibition of NF-κB, activation of p38 MAPK) | SA-β-gal, inflammatory cytokines, H&E, Ki-67, Filaggrin, Keratin 10, and Collagen 17 | [79] |
| Syringaresinol (*P. ginseng*) | Human HaCaT keratinocytes | Anti-skin aging, Antioxidant effect (autophagy) | MMP-2, MMP-9, LC-3B, ROS | [80] |
| Ginsenoside Rg1 | Bone marrow mesenchymal stem cell | Anti-senescence NRF2 and PI3K/Akt signaling | SA-β-gal, γ-H2AX, p16, p53, p21, IL-6, IL-1β | [81] |
| Ginsenoside Rg3 | Human dermal fibroblasts (HDFs) | Anti-senescence (Replicative) | SA-β-gal, ROS, sirt1/3/6,  NAD/NADH, p21, p53 | [82] |
| Ginsenoside Rb1 | C57BL/6J mice  (6-weeks old) | Anti-aging | p53, p21, Cdk2, bax, NF-κB | [83] |
| Ginsenoside Rb2 | Human dermal fibroblasts (HDFs) | Autophagy induction | SA-β-gal, p53, p21, p16, CDK4, p62 | [84] |
| Ginsenoside Re | Klotho deficient (±) mice | Upregulation of Nrf2/GPx-  1/ERK/CERB signaling | NOX, ROS, GPx, Nrf2, ERK, CERB | [85] |
| Ginsenoside Rh3 | T-HESC cells and primary murine endometrial cells | Antioxidant effect | Nrf2, HO-1, NQO1, GCLC | [86] |

**References**

[1] Zhou Q, Xu W, Yang X-W. [Chemical constituents of Chinese red ginseng]. Zhongguo Zhong Yao Za Zhi Zhongguo Zhongyao Zazhi China J Chin Mater Medica 2016;41:233–49.

[2] Qiu S, Yang W-Z, Shi X-J, Yao C-L, Yang M, Liu X, Jiang B-H, Wu W-Y, Guo D-A. A green protocol for efficient discovery of novel natural compounds: characterization of new ginsenosides from the stems and leaves of Panax ginseng as a case study. Anal Chim Acta 2015;893:65–76.

[3] Christensen LP. Ginsenosides chemistry, biosynthesis, analysis, and potential health effects. Adv Food Nutr Res 2009;55:1–99.

[4] Kim D-H. Chemical Diversity of Panax ginseng, Panax quinquifolium, and Panax notoginseng. J Ginseng Res 2012;36:1–15.

[5] Yang W, Qiao X, Li K, Fan J, Bo T, Guo D-A, Ye M. Identification and differentiation of Panax ginseng, Panax quinquefolium, and Panax notoginseng by monitoring multiple diagnostic chemical markers. Acta Pharm Sin B 2016;6:568–75.

[6] Wang H-P, Zhang Y-B, Yang X-W, Zhao D-Q, Wang Y-P. Rapid characterization of ginsenosides in the roots and rhizomes of Panax ginseng by UPLC-DAD-QTOF-MS/MS and simultaneous determination of 19 ginsenosides by HPLC-ESI-MS. J Ginseng Res 2016;40:382–94.

[7] Vinh LB, Lee Y, Han YK, Kang JS, Park JU, Kim YR, Yang SY, Kim YH. Two new dammarane-type triterpene saponins from Korean red ginseng and their anti-inflammatory effects. Bioorg Med Chem Lett 2017;27:5149–53.

[8] Wu W, Sun L, Zhang Z, Guo Y, Liu S. Profiling and multivariate statistical analysis of Panax ginseng based on ultra-high-performance liquid chromatography coupled with quadrupole-time-of-flight mass spectrometry. J Pharm Biomed Anal 2015;107:141–50.

[9] Yang H, Lee DY, Kang KB, Kim JY, Kim SO, Yoo YH, Sung SH. Identification of ginsenoside markers from dry purified extract of Panax ginseng by a dereplication approach and UPLC-QTOF/MS analysis. J Pharm Biomed Anal 2015;109:91–104.

[10] Razgonova M, Zakharenko A, Shin T-S, Chung G, Golokhvast K. Supercritical CO2 Extraction and Identification of Ginsenosides in Russian and North Korean Ginseng by HPLC with Tandem Mass Spectrometry. Mol Basel Switz 2020;25:1407.

[11] Mao Q, Bai M, Xu J-D, Kong M, Zhu L-Y, Zhu H, Wang Q, Li S-L. Discrimination of leaves of Panax ginseng and P. quinquefolius by ultra high performance liquid chromatography quadrupole/time-of-flight mass spectrometry based metabolomics approach. J Pharm Biomed Anal 2014;97:129–40.

[12] Chen W, Balan P, Popovich DG. Ginsenosides analysis of New Zealand-grown forest Panax ginseng by LC-QTOF-MS/MS. J Ginseng Res 2020;44:552–62.

[13] Lee JW, Choi B-R, Kim Y-C, Choi DJ, Lee Y-S, Kim G-S, Baek N-I, Kim S-Y, Lee DY. Comprehensive Profiling and Quantification of Ginsenosides in the Root, Stem, Leaf, and Berry of Panax ginseng by UPLC-QTOF/MS. Mol Basel Switz 2017;22:2147.

[14] Song H-H, Moon JY, Ryu HW, Noh B-S, Kim J-H, Lee H-K, Oh S-R. Discrimination of white ginseng origins using multivariate statistical analysis of data sets. J Ginseng Res 2014;38:187–93.

[15] Lee DG, Lee AY, Kim K-T, Cho EJ, Lee S. Novel Dammarane-Type Triterpene Saponins from Panax ginseng Root. Chem Pharm Bull (Tokyo) 2015;63:927–34.

[16] Wang H-Y, Hua H-Y, Liu X-Y, Liu J-H, Yu B-Y. In vitro biotransformation of red ginseng extract by human intestinal microflora: metabolites identification and metabolic profile elucidation using LC-Q-TOF/MS. J Pharm Biomed Anal 2014;98:296–306.

[17] Xie Y, Luo D, Cheng Y, Ma J, Wang Y, Liang Q, Luo G. Steaming-induced chemical transformations and holistic quality assessment of red ginseng derived from Panax ginseng by means of HPLC-ESI-MS/MS(n)-based multicomponent quantification fingerprint. J Agric Food Chem 2012;60:8213–24.

[18] Shi X, Yang W, Huang Y, Hou J, Qiu S, Yao C, Feng Z, Wei W, Wu W, Guo D. Direct screening of malonylginsenosides from nine Ginseng extracts by an untargeted profiling strategy incorporating in-source collision-induced dissociation, mass tag, and neutral loss scan on a hybrid linear ion-trap/Orbitrap mass spectrometer coupled to ultra-high performance liquid chromatography. J Chromatogr A 2018;1571:213–22.

[19] Yuan J, Chen Y, Liang J, Wang C-Z, Liu X, Yan Z, Tang Y, Li J, Yuan C-S. Component analysis and target cell-based neuroactivity screening of Panax ginseng by ultra-performance liquid chromatography coupled with quadrupole-time-of-flight mass spectrometry. J Chromatogr B Analyt Technol Biomed Life Sci 2016;1038:1–11.

[20] Chu C, Xu S, Li X, Yan J, Liu L. Profiling the ginsenosides of three ginseng products by LC-Q-TOF/MS. J Food Sci 2013;78:C653-659.

[21] Jia X, Hu C, Zhu X, Yuan Y, Zhou Y. Simultaneous Determination of 25 Ginsenosides by UPLC-HRMS via Quantitative Analysis of Multicomponents by Single Marker. Int J Anal Chem 2021;2021:9986793.

[22] Liang Z, Chen Y, Xu L, Qin M, Yi T, Chen H, Zhao Z. Localization of ginsenosides in the rhizome and root of Panax ginseng by laser microdissection and liquid chromatography-quadrupole/time of flight-mass spectrometry. J Pharm Biomed Anal 2015;105:121–33.

[23] Fuzzati N. Analysis methods of ginsenosides. J Chromatogr B Analyt Technol Biomed Life Sci 2004;812:119–33.

[24] Wang H-P, Zhang Y-B, Yang X-W, Yang X-B, Xu W, Xu F, Cai S-Q, Wang Y-P, Xu Y-H, Zhang L-X. High-Performance Liquid Chromatography with Diode Array Detector and Electrospray Ionization Ion Trap Time-of-Flight Tandem Mass Spectrometry to Evaluate Ginseng Roots and Rhizomes from Different Regions. Mol Basel Switz 2016;21:603.

[25] Li W, Zheng L, Ma X, Xia J, Sheng J, Ge P, Yuan Y, Fan Y, Zhou Y. The sugar moiety in protopanaxadiol ginsenoside affects its ability to target glucocorticoid receptor to regulate lipid metabolism. Bioorganic Chem 2024;153:107885.

[26] Ali MY, Zaib S, Jannat S, Khan I, Rahman MM, Park SK, Chang MS. Inhibition of Aldose Reductase by Ginsenoside Derivatives via a Specific Structure Activity Relationship with Kinetics Mechanism and Molecular Docking Study. Mol Basel Switz 2022;27:2134.

[27] Quan K, Liu Q, Wan J-Y, Zhao Y-J, Guo R-Z, Alolga RN, Li P, Qi L-W. Rapid preparation of rare ginsenosides by acid transformation and their structure-activity relationships against cancer cells. Sci Rep 2015;5:8598.

[28] Lee J-H, Choi S-H, Lee B-H, Yoon I-S, Shin T-J, Pyo MK, Lee S-M, Rhim H, Park MH, Park TY, et al. Modifications of aliphatic side chain of 20(S)-ginsenoside RG3 cause an enhancement or loss of brain Na+ channel current inhibitions. Biol Pharm Bull 2008;31:480–6.

[29] Kitagawa S, Takahashi T, Nabekura T, Tachikawa E, Hasegawa H. Inhibitory effects of ginsenosides and their hydrolyzed metabolites on daunorubicin transport in KB-C2 cells. Biol Pharm Bull 2007;30:1979–81.

[30] Liu J, Shimizu K, Yu H, Zhang C, Jin F, Kondo R. Stereospecificity of hydroxyl group at C-20 in antiproliferative action of ginsenoside Rh2 on prostate cancer cells. Fitoterapia 2010;81:902–5.

[31] Dong H, Bai L-P, Wong VKW, Zhou H, Wang J-R, Liu Y, Jiang Z-H, Liu L. The in vitro structure-related anti-cancer activity of ginsenosides and their derivatives. Mol Basel Switz 2011;16:10619–30.

[32] Feng R, Liu J, Wang Z, Zhang J, Cates C, Rousselle T, Meng Q, Li J. The structure-activity relationship of ginsenosides on hypoxia-reoxygenation induced apoptosis of cardiomyocytes. Biochem Biophys Res Commun 2017;494:556–68.

[33] Setiawan T, Sari IN, Wijaya YT, Julianto NM, Muhammad JA, Lee H, Chae JH, Kwon HY. Cancer cachexia: molecular mechanisms and treatment strategies. J Hematol OncolJ Hematol Oncol 2023;16:54.

[34] Neshan M, Tsilimigras DI, Han X, Zhu H, Pawlik TM. Molecular Mechanisms of Cachexia: A Review. Cells 2024;13:252.

[35] Pang X, Zhang P, Chen X, Liu W. Ubiquitin-proteasome pathway in skeletal muscle atrophy. Front Physiol 2023;14:1289537.

[36] Compton SLE, Heymsfield SB, Brown JC. Nutritional Mechanisms of Cancer Cachexia. Annu Rev Nutr 2024;44:77–98.

[37] Ispoglou T, McCullough D, Windle A, Nair S, Cox N, White H, Burke D, Kanatas A, Prokopidis K. Addressing cancer anorexia-cachexia in older patients: Potential therapeutic strategies and molecular pathways. Clin Nutr Edinb Scotl 2024;43:552–66.

[38] Pandey S, Bradley L, Del Fabbro E. Updates in Cancer Cachexia: Clinical Management and Pharmacologic Interventions. Cancers 2024;16:1696.

[39] Wijaya YT, Setiawan T, Sari IN, Nah S-Y, Kwon HY. Amelioration of muscle wasting by gintonin in cancer cachexia. Neoplasia N Y N 2021;23:1307–17.

[40] Kim H-J, Kim SJ, Woo C-W, Kim S-T, Im M, Park SK, Kim J-Y, Yoo HJ, Woo D-C, Kim JK. Treatment of chemotherapy-induced cachexia with BST204: a multimodal validation study. Metabolomics Off J Metabolomic Soc 2021;17:36.

[41] Lu S, Zhang Y, Li H, Zhang J, Ci Y, Han M. Ginsenoside Rb1 can ameliorate the key inflammatory cytokines TNF-α and IL-6 in a cancer cachexia mouse model. BMC Complement Med Ther 2020;20:11.

[42] Kim A, Im M, Ma JY. A novel herbal formula, SGE, induces endoplasmic reticulum stress-mediated cancer cell death and alleviates cachexia symptoms induced by colon-26 adenocarcinoma. Oncotarget 2018;9:16284–96.

[43] Kim J, Choi H-J, Seo D, Lee S-A, Heo JB, Baek DH, Lee W, Song GY. Steamed Ginseng Berry Powder Ameliorates Skeletal Muscle Atrophy via Myogenic Effects. J Microbiol Biotechnol 2024;34:157–66.

[44] Jeong J-S, Kim J-W, Kim J-H, Kim C-Y, Ko J-W, Kim T-W. Korean red ginseng suppresses mitochondrial apoptotic pathway in denervation-induced skeletal muscle atrophy. J Ginseng Res 2024;48:52–8.

[45] Kim R, Kim JW, Choi H, Oh J-E, Kim TH, Go G-Y, Lee S-J, Bae G-U. Ginsenoside Rg5 promotes muscle regeneration via p38MAPK and Akt/mTOR signaling. J Ginseng Res 2023;47:726–34.

[46] Cho D-E, Choi G-M, Lee Y-S, Hong J-P, Yeom M, Lee B, Hahm D-H. Long-term administration of red ginseng non-saponin fraction rescues the loss of skeletal muscle mass and strength associated with aging in mice. J Ginseng Res 2022;46:657–65.

[47] Oh H-J, Jin H, Lee B-Y. The non-saponin fraction of Korean Red Ginseng ameliorates sarcopenia by regulating immune homeostasis in 22-26-month-old C57BL/6J mice. J Ginseng Res 2022;46:809–18.

[48] Jin H, Oh H-J, Nah S-Y, Lee B-Y. Gintonin-enriched fraction protects against sarcopenic obesity by promoting energy expenditure and attenuating skeletal muscle atrophy in high-fat diet-fed mice. J Ginseng Res 2022;46:454–63.

[49] Go G-Y, Jo A, Seo D-W, Kim W-Y, Kim YK, So E-Y, Chen Q, Kang J-S, Bae G-U, Lee S-J. Ginsenoside Rb1 and Rb2 upregulate Akt/mTOR signaling-mediated muscular hypertrophy and myoblast differentiation. J Ginseng Res 2020;44:435–41.

[50] Xiang Y-Z, Shang H-C, Gao X-M, Zhang B-L. A comparison of the ancient use of ginseng in traditional Chinese medicine with modern pharmacological experiments and clinical trials. Phytother Res PTR 2008;22:851–8.

[51] Zheng X, Liang Y, Kang A, Ma S-J, Xing L, Zhou Y-Y, Dai C, Xie H, Xie L, Wang G-J, et al. Peripheral immunomodulation with ginsenoside Rg1 ameliorates neuroinflammation-induced behavioral deficits in rats. Neuroscience 2014;256:210–22.

[52] Davies RF. Long- and short-term regulation of feeding patterns in the rat. J Comp Physiol Psychol 1977;91:574–85.

[53] Kang M, Yoshimatsu H, Oohara A, Kurokawa M, Ogawa R, Sakata T. Ginsenoside Rg1 modulates ingestive behavior and thermal response induced by interleukin-1β in rats. Physiol Behav 1995;57:393–6.

[54] Raghavendran HRB, Rekha S, Shin J-W, Kim H-G, Wang J-H, Park H-J, Choi M-K, Cho J-H, Son C-G. Effects of Korean ginseng root extract on cisplatin-induced emesis in a rat-pica model. Food Chem Toxicol 2011;49:215–21.

[55] Gao Y-J, Wu X-P, Ye B-B, Dai Y-Z, Zhao Y-Z, Liao X-X, Nie K. Liu-Jun-Zi decoction alleviates chemotherapy-induced anorexia by regulating gut microbiota and TLR4/MyD88/NF-κB p65 signaling pathway. Tradit Med Res 2024;9:47.

[56] Dai Y, Hu W, Han J, Zhao Y, Wu X, Liao X, Nie K. Liujunzi decoction attenuates cisplatin-induced anorexia in rats via inhibiting PERK/eIF2α/ATF4/CHOP pathway and GDF15/GFRAL expression. J Holist Integr Pharm 2024;5:113–20.

[57] Goswami C, Dezaki K, Wang L, Inui A, Seino Y, Yada T. Ninjin-yoeito activates ghrelin-responsive and unresponsive NPY neurons in the arcuate nucleus and counteracts cisplatin-induced anorexia. Neuropeptides 2019;75:58–64.

[58] Ko M-H, Song S-Y, Ha S-J, Lee JY, Yoon SW, Park J-H, Park S-J, Yoo H-S. Efficacy and Safety of Yukgunja-Tang for Patients with Cancer-related Anorexia: A Randomized, Controlled Trial, Pilot Study. Integr Cancer Ther 2021;20:15347354211019107.

[59] Cheon C, Park S, Park YL, Huang C-W, Ko Y, Jang B-H, Shin Y-C, Ko S-G. Sipjeondaebo-tang in patients with cancer with anorexia: a protocol for a pilot, randomised, controlled trial. BMJ Open 2016;6:e011212.

[60] Choi YK, Jung KY, Woo S-M, Yun YJ, Jun C-Y, Park JH, Shin YC, Cho S-G, Ko S-G. Effect of Sipjeondaebo-Tang on Cancer-Induced Anorexia and Cachexia in CT-26 Tumor-Bearing Mice. Mediators Inflamm 2014;2014:1–10.

[61] Cho I-H, Son GM, Choi J-Y, Shin HK, Kim S. Bojungikgi-tang for Anorexia in Colorectal Cancer Patients after Curative Resection and Chemotherapy: A Single-arm, Open-label, Single-center Trial. J Korean Med 2024;45:150–67.

[62] Min D, Kim B, Ko S-G, Kim W. Effect and Mechanism of Herbal Medicines on Cisplatin-Induced Anorexia. Pharm Basel Switz 2022;15:208.

[63] Kang A, Xie T, Zhu D, Shan J, Di L, Zheng X. Suppressive Effect of Ginsenoside Rg3 against Lipopolysaccharide-Induced Depression-Like Behavior and Neuroinflammation in Mice. J Agric Food Chem 2017;65:6861–9.

[64] Yennurajalingam S, Reddy A, Tannir NM, Chisholm GB, Lee RT, Lopez G, Escalante CP, Manzullo EF, Frisbee Hume S, Williams JL, et al. High-Dose Asian Ginseng (Panax Ginseng) for Cancer-Related Fatigue: A Preliminary Report. Integr Cancer Ther 2015;14:419–27.

[65] Phung HM, Jang D, Trinh TA, Lee D, Nguyen QN, Kim C-E, Kang KS. Regulation of appetite-related neuropeptides by Panax ginseng: A novel approach for obesity treatment. J Ginseng Res 2022;46:609–19.

[66] Takayama S, Ishii T. Kampo medicine bukuryoingohangekobokuto and ninjin’yoeitoyoeito as supportive care for management of anorexia and tightness of the esophagus in advanced esophageal cancer: A case report. Explore N Y N 2023;19:256–9.

[67] Zhang X, Qiu H, Li C, Cai P, Qi F. The positive role of traditional Chinese medicine as an adjunctive therapy for cancer. Biosci Trends 2021;15:283–98.

[68] Zhu L, Zhang J, Hu Y, Zhang Y, Wang S, Wang R, Yuan Q, Luo Y, Yuan D, He Y, et al. Total saponins from Panax Japonicus regulate the ferritin heavy chain to reduce inflammation in aging adipose tissue by mediating iron metabolism. J Ginseng Res 2025;49:134–44.

[69] Kang M, Park S, Son S-R, Noh Y, Jang DS, Lee S. Anti-Aging and Anti-Inflammatory Effects of Compounds from Fresh Panax ginseng Roots: A Study on TNF-α/IFN-γ-Induced Skin Cell Damage. Mol Basel Switz 2024;29:5479.

[70] Wang L, Qiao P, Ouyang Z, Li D, Zheng J, Wang G, Wang F. Ginseng volatile oil prolongs the lifespan and healthspan of Caenorhabditis elegans. Biogerontology 2022;23:485–97.

[71] Kim T-H, Kim J-Y, Bae J, Kim Y-M, Won M-H, Ha K-S, Kwon Y-G, Kim Y-M. Korean Red ginseng prevents endothelial senescence by downregulating the HO-1/NF-κB/miRNA-155-5p/eNOS pathway. J Ginseng Res 2021;45:344–53.

[72] Kang H, Lim JW, Kim H. Inhibitory effect of Korean Red Ginseng extract on DNA damage response and apoptosis in Helicobacter pylori-infected gastric epithelial cells. J Ginseng Res 2020;44:79–85.

[73] Wang J, Zhou Y, Yu Y, Wang Y, Xue D, Zhou Y, Li X. A ginseng-derived rhamnogalacturonan I (RG-I) pectin promotes longevity via TOR signalling in Caenorhabditis elegans. Carbohydr Polym 2023;312:120818.

[74] Lee Y, Oh S. Administration of red ginseng ameliorates memory decline in aged mice. J Ginseng Res 2015;39:250–6.

[75] Zhu N, Xu M-H, Li Y. Bioactive Oligopeptides from Ginseng (Panax ginseng Meyer) Suppress Oxidative Stress-Induced Senescence in Fibroblasts via NAD+/SIRT1/PGC-1α Signaling Pathway. Nutrients 2022;14:5289.

[76] Lee S-J, Lee D-Y, O’Connell JF, Egan JM, Kim Y. Black Ginseng Ameliorates Cellular Senescence via p53-p21/p16 Pathway in Aged Mice. Biology 2022;11:1108.

[77] Hou W, Pei J, Wang Y, Zhang J, Zheng H, Cui R. Anti-ageing effects of red ginseng on female Drosophila melanogaster. J Cell Mol Med 2020;24:3751–5.

[78] Nam YH, Jeong SY, Kim YH, Rodriguez I, Nuankaew W, Bhawal UK, Hong BN, Kang TH. Anti-aging effects of Korean Red Ginseng (KRG) in differentiated embryo chondrocyte (DEC) knockout mice. J Ginseng Res 2021;45:183–90.

[79] Kim AY, Jung J-Y, Kim D, Cho JH, Hong YD, Kim H-J, Cho S-Y. Compound K suppresses epidermal aging induced by IL-17A treatment and UVB irradiation. J Ginseng Res 2025;49:326–30.

[80] Choi W, Kim HS, Park SH, Kim D, Hong YD, Kim JH, Cho JY. Syringaresinol derived from Panax ginseng berry attenuates oxidative stress-induced skin aging via autophagy. J Ginseng Res 2022;46:536–42.

[81] Wang Z, Wang L, Jiang R, Li C, Chen X, Xiao H, Hou J, Hu L, Huang C, Wang Y. Ginsenoside Rg1 prevents bone marrow mesenchymal stem cell senescence via NRF2 and PI3K/Akt signaling. Free Radic Biol Med 2021;174:182–94.

[82] Yang K-E, Jang H-J, Hwang I-H, Hong EM, Lee M-G, Lee S, Jang I-S, Choi J-S. Stereoisomer-specific ginsenoside 20(S)-Rg3 reverses replicative senescence of human diploid fibroblasts via Akt-mTOR-Sirtuin signaling. J Ginseng Res 2020;44:341–9.

[83] Yu S, Xia H, Guo Y, Qian X, Zou X, Yang H, Yin M, Liu H. Ginsenoside Rb1 retards aging process by regulating cell cycle, apoptotic pathway and metabolism of aging mice. J Ethnopharmacol 2020;255:112746.

[84] Yang KE, Nam S-B, Jang M, Park J, Lee G-E, Cho Y-Y, Jang B-C, Lee C-J, Choi J-S. Ginsenoside Rb2 suppresses cellular senescence of human dermal fibroblasts by inducing autophagy. J Ginseng Res 2023;47:337–46.

[85] Nguyen BT, Shin E-J, Jeong JH, Sharma N, Nah SY, Ko SK, Byun JK, Lee Y, Lei XG, Kim D-J, et al. Ginsenoside Re attenuates memory impairments in aged Klotho deficient mice via interactive modulations of angiotensin II AT1 receptor, Nrf2 and GPx-1 gene. Free Radic Biol Med 2022;189:2–19.

[86] Wang X-M, She C, Li Q, Zhang D, Xu J-X, Li M-H, Li P, Xu H-B. Ginsenoside Rh3 activates Nrf2 signaling and protects endometrial cells from oxygen and glucose deprivation-reoxygenation. Aging 2020;12:6109–19.
